# The independent prognostic nomogram models for primary and recurrent retroperitoneal liposarcoma: a population-based cohort study

**DOI:** 10.3389/fmed.2025.1642820

**Published:** 2025-08-06

**Authors:** Huan Deng, Zhenhua Lu, Yajie Wang, Lin Xiao, Yisheng Pan

**Affiliations:** ^1^Department of Gastrointestinal Surgery, Peking University First Hospital, Beijing, China; ^2^Department of General Surgery, The First Medical Center, Chinese People’s Liberation Army General Hospital, Beijing, China; ^3^Department of Gastrointestinal Surgery, Key Laboratory of Carcinogenesis and Translational Research (Ministry of Education), Peking University Cancer Hospital & Institute, Beijing, China

**Keywords:** retroperitoneal liposarcoma, primary, recurrent, prognosis, nomogram

## Abstract

**Purpose:**

The aim of this study was to screen and establish independent prognostic models for primary and recurrent retroperitoneal liposarcoma (RLS).

**Methods:**

A total of 2,429 patients confirmed to have RLS were extracted from the Surveillance, Epidemiology and End Results (SEER) database. The 245 patients collected from the same period at First Medical Center, Chinese People Liberation Army General Hospital (CPLAGH), were used for external validations. Nomogram were built on the basis of clinical practicability, univariate and multivariate Cox analyses.

**Results:**

After performing a stepwise analysis, the simplified predictive models for primary RLS were primarily based on tumor size (median size, 162 mm [range, 90–230], *p* < 0.001) and pathological subtypes (WDL vs. DDL, hazard ratio [HR] = 2.11; 95% confidence interval [CI] = 1.71–2.61; *p* < 0.001), both of which can be readily obtained in outpatient settings. In contrast, TNM stage (HR = 2.18; 95% CI = 1.49–3.20; *p* < 0.001), an important postoperative prognostic factor, emerged as a significant predictor for recurrent RLS. The area under the time-dependent receiver operating characteristic curve (time-dependent AUC) and the concordance index (C-index) for overall survival (OS) and cancer-specific survival (CSS) models both approached 0.75 in both training and validation cohorts. Moreover, calibration curves and decision curve analysis (DCA) demonstrated that the validated models were not only reliable but also clinically applicable.

**Conclusion:**

We have developed efficient and independent models for both primary and recurrent RLS. These models will provide invaluable clinical guidance, aiding in prognostication and facilitating personalized therapeutic decision-making.

## Introduction

Retroperitoneal liposarcoma (RLS) is a type of rare malignant tumor arising in the retroperitoneum. According to official reports and previous studies, the incidence of RLS is approximately 0.07 to 0.2% among all tumors and approximately 12 to 40% among all liposarcomas (1). RLS can be further divided into the following four pathological subtypes: well-differentiated liposarcoma (WDL), dedifferentiated liposarcoma (DDL), myxoid cell liposarcoma (MLS), and pleomorphic liposarcoma (PLS) (2). DDL and WDL have extremely similar morphological and genetic mutation origin characteristics and are the most common subtypes of RLS (3). RLS grows covertly in the retroperitoneal regions without typical clinical symptoms, making it difficult to detect in daily life.

The prognosis of RLS is associated with many factors, such as therapeutic methods and the degree of pathological differentiation (4–6). Although great advancements in treatment have been achieved in recent years, the prognosis of patients with recurrent RLS is not optimal. Surgical resection is currently the main treatment for this disease (7). Resection margins and strategies are considered important factors affecting local recurrence and overall survival (5). However, some patients experience recurrence after radical resection of their primary tumor. Hence, a deeper understanding of the pathogenesis and treatment of RLS is needed. Studies have shown that the degree of differentiation of RLS is closely related to the prognosis, and poor differentiation is associated with local recurrence and distant metastasis (6, 8). The differ genetic alterations of each pathological subtype of RLS, resulting in significant differences in prognosis (4, 9, 10).

Numerous studies have explored the clinical and pathological characteristics of recurrent RLS. Many RLS patients experience postoperative recurrence. Patients who experience a recurrence usually have a poor prognosis (11). During clinical practice, we found that patients with multifocal tumors or incomplete resection margins were more likely to experience recurrence (12, 13). Radical resection of tumors improves the survival outcomes of patients with RLS. However, the large volume of tumors may limit the ability of surgeons to achieve perfect radical resection. In addition, RLS can invade important blood vessels and organs in the deep retroperitoneal regions and usually requires removal of the kidney and sometimes other organs to achieve curative treatment (14, 15). These factors are therapeutic obstacles and associated with poor outcomes of patients with RLS.

The accuracy and efficient prognostic models are very important for guiding the clinical treatment and management of RLS. Some previous studies have provided predictive models for overall survival (OS) or cancer specific survival (CSS) (16, 17). However, those models included a relatively small number of cases and did not clarify the prognostic differences between primary and recurrent RLS. We used the SEER database to enroll a larger cohort of patients with RLS, including primary and recurrent patients, and independently built reliable models. Elucidating the effects of independent models on the prognosis of RLS would be helpful for the treatment of RLS.

This study is one of the largest retrospective cohort to compare differences between primary and recurrent RLS. We analyzed the basic clinicopathological features of RLS and investigated the important prognostic factors for this subset of patients. The internal and external validated models could provide reliable suggestions for both doctors and patients. This study provides evidence to support the individualized clinical management of RLS.

## Materials and methods

### Study design and participants

Data used in this study were extracted from two sources. The first source was the SEER database provided by the National Cancer Institute’s SEER*Stat software version 8.4.3^1^. The second source was RLS patients treated at the First Medical Center, CPLAGH from 2000 to 2020. This study was approved by the Protection of Human Subjects Committee of the CPLAGH. The cases of second source served as external validations. The screening of patients with RLS in the SEER database is shown in Supplementary Figure S1. However, cases with distant organ metastasis were missing from the second source. Patients who received adjuvant radiotherapy or chemotherapy were also not enrolled in the second source.

### Outcomes and definitions

OS was defined as the time from randomization or treatment to death from any cause, and CSS was defined as the time from randomization or treatment until death due to the specific cancer. CSS exclude deaths caused by other reasons. Primary RLS was defined as the first time RLS tumors were diagnosed. Recurrent RLS was defined as a RLS tumor that relapsed at least once since the initial diagnosis. The occurrence pattern refers to the overall description of different disease states and mechanisms of RLS, encompassing both primary and recurrent conditions.

### Statistical analysis

Potential prognostic variables were identified using univariate Cox regression analysis. Variables with multicollinearity, as indicated by a variance inflation factor (VIF) greater than 4, were excluded. The remaining variables were then included in the final multivariate Cox regression analysis. The Cox regression models were constructed via the survival coxph function of the R package. To evaluate the statistical significance of the differences observed between these groups, the log-rank test was applied. The data were analyzed and visualized using R software (Version 4.3.1). A two-sided *p* value <0.05 was considered statistically significant.

## Results

### Characteristics of patients and disease

**Figure 1:**
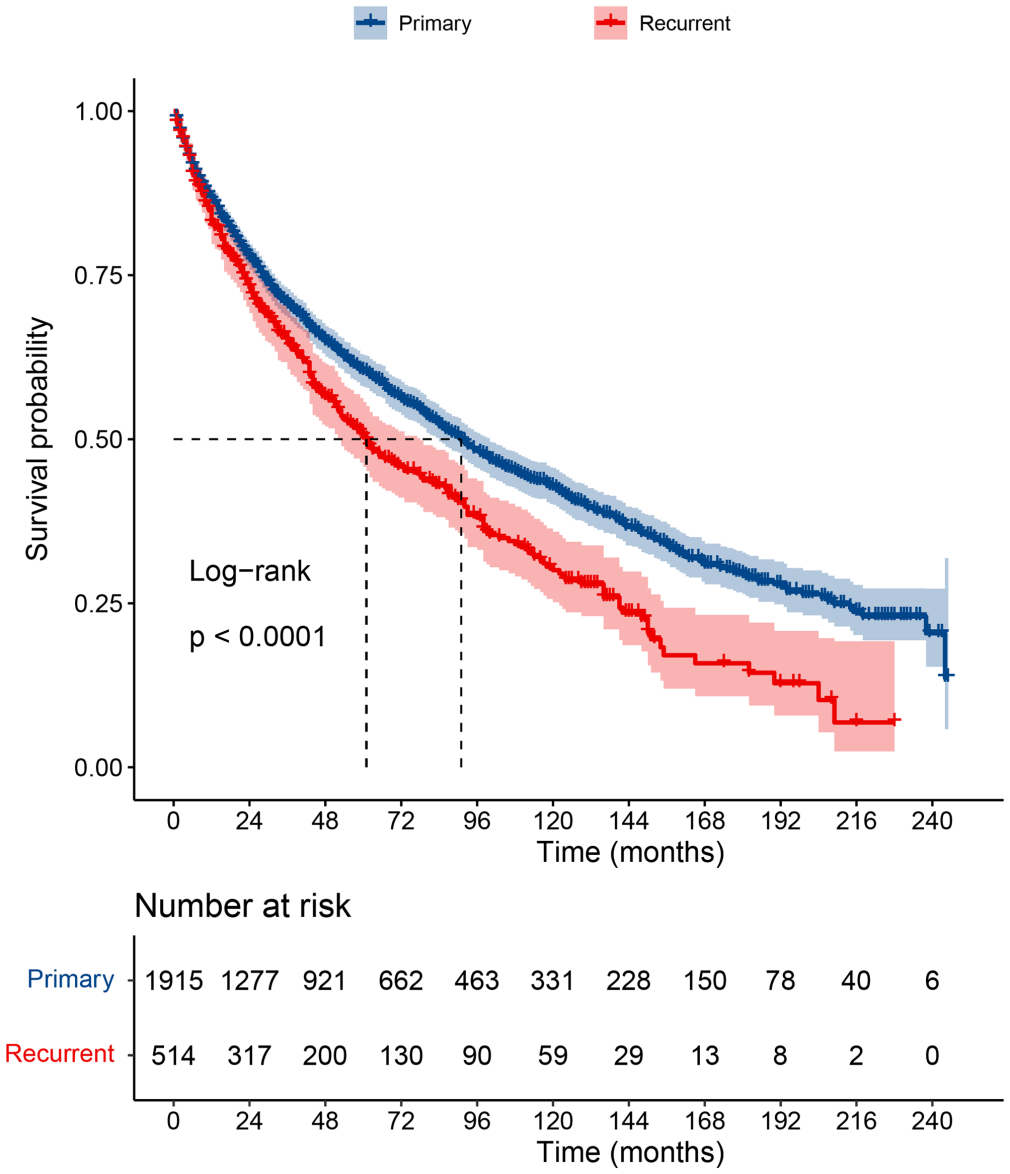
The Kaplan-Meier survival curves for patients with primary RLS or recurrent RLS. RLS, retroperitoneal liposarcoma.

A total of 2,429 patients with RLS were ultimately included in this study. Among them, 1912 patients were primary case (Sequence Number in SEER: 00 and 01), and 514 patients were recurrent case (Sequence Number in SEER: 02). The demographic and clinical features of these patients are summarized and compared in Table 1. Patients with recurrent RLS had poorer survival outcomes than those with primary RLS (median OS, primary 91 months vs. recurrent 61 months, *p* < 0.05, Figure 1). The above results revealed many differences in the clinical characteristics and survival outcomes between primary and recurrent cases. Hence, we built independent prognostic models for the two types of patients.

**Table 1:** Comparison of demographic, clinical, and pathological characteristics of primary and recurrent RLS patients included.

Variable	Primary, *N* = 1,915	Recurrence, *N* = 514	*p*-value
Sex			<0.001
Female	867 (45%)	189 (37%)	
Male	1,048 (55%)	325 (63%)	
Age	63 (53–71)	68 (60–76)	<0.001
Income			0.5
High	889 (46%)	253 (49%)	
Middle	699 (37%)	175 (34%)	
Low	327 (17%)	86 (17%)	
City			0.2
Metropolitan	1,721 (90%)	451 (88%)	
Nonmetropolitan	194 (10%)	63 (12%)	
Tumor size	200 (130, 280)	161 (90, 240)	<0.001
T			<0.001
T1	115 (6.0%)	51 (9.9%)	
T2	210 (11%)	105 (20%)	
T3	333 (17%)	88 (17%)	
T4	1,257 (66%)	270 (53%)	
*N*			0.9
N0	1,512 (79%)	411 (80%)	
N1	37 (1.9%)	10 (1.9%)	
Unknown	366 (19%)	93 (18%)	
M			0.062
M0	1,452 (76%)	410 (80%)	
M1	102 (5.3%)	16 (3.1%)	
Unknown	361 (19%)	88 (17%)	
TNM stage			0.26
Stage 1 (I-II)	910 (48%)	265 (52%)	
Stage 2 (III-IV)	587 (31%)	148 (29%)	
Unknown	418 (22%)	101 (20%)	
Grade			0.5
Well differentiated	840 (44%)	210 (41%)	
Moderately differentiated	151 (7.9%)	36 (7.0%)	
Poorly differentiated	256 (13%)	77 (15%)	
Undifferentiated	327 (17%)	88 (17%)	
Unknown	341 (18%)	103 (20%)	
Chemotherapy			0.006
No/Unknown	1,662 (87%)	469 (91%)	
Yes	253 (13%)	45 (8.8%)	
Pathological subtypes			0.5
WDL	773 (40%)	197 (38%)	
MLS	113 (5.9%)	26 (5.1%)	
PLS	49 (2.6%)	9 (1.8%)	
DDL	824 (43%)	233 (45%)	
Liposarcoma, NOS	156 (8.1%)	49 (9.5%)	
Surgery			0.5
No surgery	200 (10%)	61 (12%)	
Partial surgical	745 (39%)	206 (40%)	
Total surgical	970 (51%)	247 (48%)	
Overall survival months	44 (16–93)	33 (13–73)	<0.001

RLS retroperitoneal liposarcoma, WDL Well-differentiated liposarcoma, MLS Myxoid cell liposarcoma, PLS Pleomorphic liposarcoma, DDL Dedifferentiated liposarcoma. Categorical data are expressed as frequencies (percentages) and continous variables are expressed as the median (Q1-Q3).

In the primary and recurrent cohorts, the median ages of the patients were 63 (IQR: 53–71) and 68 (IQR: 60–76) years, respectively. We further randomly divided the cohorts into a training cohort and a validation cohort at a ratio of 7:3. In the primary RLS cohort, a total of 1,340 patients were included in the training set, and 575 patients were included in the internal validation set (Supplementary Table S1). Meanwhile, in the recurrent cohort, 359 patients were assigned to the training set, and 155 patients were assigned to the internal validation set (Supplementary Table S2). The divided cohorts were comparable in terms of demographic and clinical features (*p* > 0.05).

### Survival predictive factor screening and nomogram model establishment

In this study, we conducted univariate and multivariate Cox analyses on variables for the prediction of OS and CSS of RLS patients. According to the stepwise regression results, those factors that were significant in the univariate analysis were further analyzed via multivariate analysis. Factors with VIF values > 4, indicating that collinearity existed between the screened variables, were excluded from the multivariate analysis (18).

**Figure 2:**
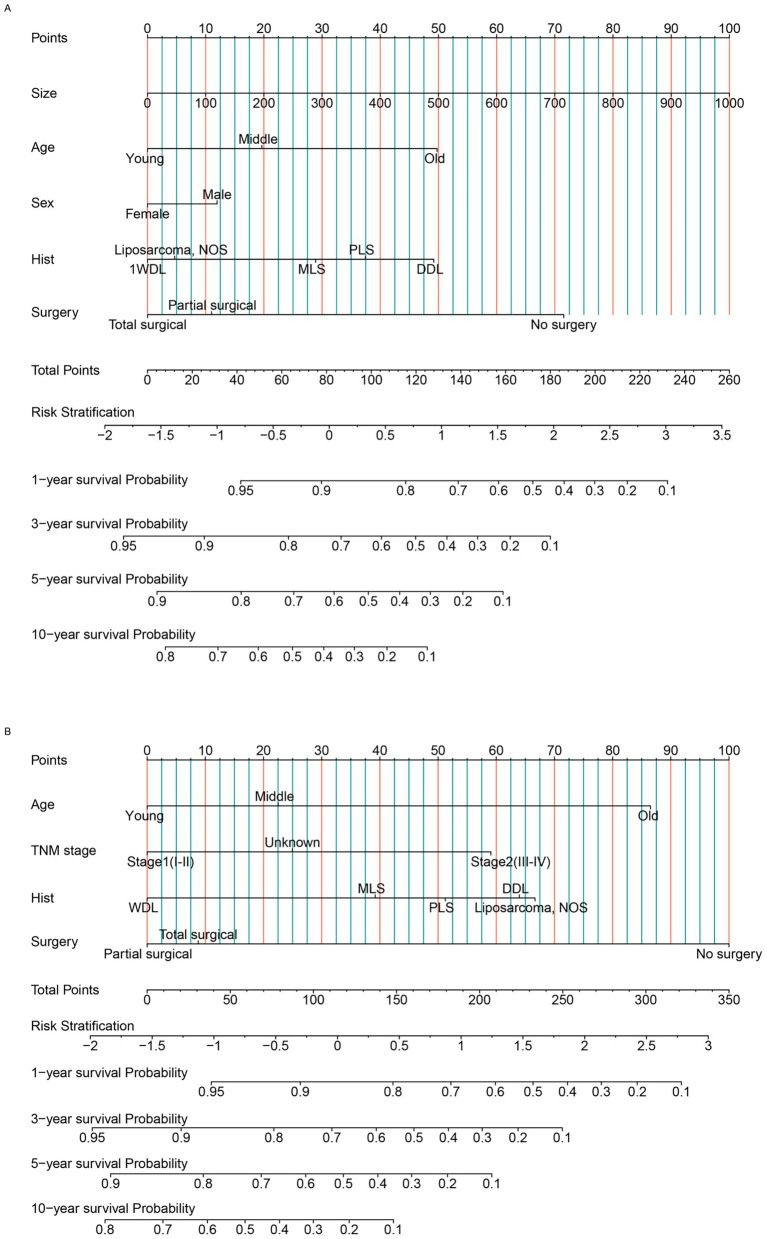
The nomogram model was built to predict the overall survival status of patients with RLS. **(A)** The nomogram for primary patients. **(B)** The nomogram for recurrent patients. RLS, retroperitoneal liposarcoma; WDL, well-differentiated liposarcoma; DDL, dedifferentiated liposarcoma; MLS, myxoid cell liposarcoma; PLS, pleomorphic liposarcoma; liposarcoma NOS: Unable to determine the specific subtype. AJCC TNM stage, Unknown: unable to determine the specific stage. “Hist”: pathological subtypes. Age, Young: ≦ 60 years old, Middle: 60-75 years old, Old: ≧ 75 years old (Classification criteria: according to the distribution features and previous studies).

In the primary RLS cohort, age, sex, residence, tumor size, N stage, M stage, TNM stage, chemotherapy, pathological subtype, histological grade and surgical method were significant in the univariate analysis (*p* < 0.05). Owing to the effect of collinearity, N stage (VIF = 22), M stage (VIF = 26) and histological grade (VIF = 7.5) were excluded from the multivariate analysis (Supplementary Table S3). The chemotherapy indicator was excluded from the multivariate analysis because this factor was missing for most patients. We ultimately included the remaining 7 factors in the multivariate analysis. Age, tumor size, sex, TNM stage, pathological subtype and surgical method were important indicators for primary RLS. For patients with newly diagnosed primary RLS who have not yet undergone surgery, a practical preoperative nomogram model will be more meaningful to provide guidance for both clinicians and patients in the pretreatment phase. However, TNM stage is a factor that is difficult to acquire in the outpatient phase, so, we excluded it from the nomogram model. Hence, we used the acquirable clinical indicators of age, sex, tumor size, pathological subtype and proposed surgical method to construct a preoperative OS nomogram model (Figure 2A). The C-index of the nomogram model for OS prediction was approximately 0.71. Patients need only simple imaging examinations and tumor biopsies in the outpatient department. Then, this model can be used for preliminary prognostication and surgical mode selection. Similarly, we constructed a CSS nomogram model for patients with primary RLS (Supplementary Table S4, Supplementary Figure S2). The C-index of the nomogram model for predicting CSS was approximately 0.74.

For patients with recurrent RLS, because they have undergone previous treatment, the acquirable factors are different from those of primary patients. We found that age, N stage, M stage, TNM stage, occurrence pattern, chemotherapy, pathological subtype, histological grade and surgical method were significant in the univariate analysis (*p* < 0.05). Owing to collinearity, N stage (VIF = 14), M stage (VIF = 17) and histological grade (VIF = 11) were not included in the multivariate analysis (Supplementary Table S5). Finally, we included age, TNM stage, pathological subtype and surgical method in the multivariate analysis to construct the OS nomogram model (Figure 2B). These factors, including clinical factors and postoperative indicators, are more practical and useful for recurrent patients. The C-index of the nomogram model for OS prediction was approximately 0.74. Similarly, we established a CSS nomogram model for patients with recurrent RLS. According to the multivariate analysis, we found that TNM stage was an insignificant factor for CSS prediction (Supplementary Table S6). Age, occurrence pattern, pathological subtype and surgical method were ultimately included in the nomogram model for CSS (Supplementary Figure S3). According to the nomogram model, the survival outcomes of patients with non-localized recurrence are poorer. This revealed that the occurrence pattern (“SEER stage” in the SEER database) was important for predicting the CSS. The C-index of the nomogram model for CSS prediction was approximately 0.75.

### Validation of the constructed nomogram models

**Figure 3:**
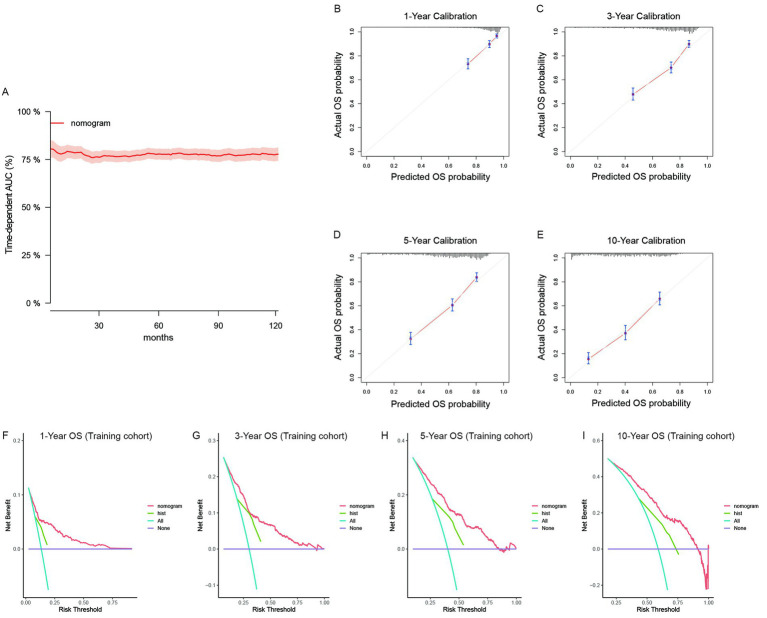
The validation of the OS predictive nomogram for primary RLS. **(A)** Time-dependent AUC of using the nomogram to predict overall survival probability within 10 years in the training cohort. The 95% confidence interval was calculated by using the bootstrapping cross-validation method. **(B–E)** Calibration curves of 1-year, 3-year, 5-year and 10-year OS in the training cohort. **(F–I)** Decision curve analysis of the nomogram and pathologic subtypes (hist) for the survival prediction within 10 years.

The nomogram models were established on the basis of multivariate Cox analyses and clinical practicability. For the primary cohort, the model had good predictive value for OS. The total score was calculated on the basis of the individual scores determined using the nomogram model; most patients in this study had total risk points ranging from 50 to 103. The time-dependent AUC was greater than 0.75 for the prediction of OS within 10 years in the training cohort (Figure 3A) and two validation cohorts (Supplementary Figures S4A,B). These results indicated favorable discrimination of the nomogram models. The calibration curves of the model illustrated better consistency between the predicted and observed OS probabilities in both the training (Figures 3B–E) and validation cohorts (Supplementary Figures S4C–I). The clinical benefits of the nomogram model were shown by DCA curves in this study. Compared with the predictive value of pathological subtypes, DCA curves revealed that the nomogram could better predict 10-year OS, as it added more net benefits for almost all threshold probabilities in both the training (Figures 3F–I) and validation cohorts (Supplementary Figures S4J–P). Similarly, we validated the discrimination, consistency and clinical benefits of the CSS nomogram model using the training cohort (Supplementary Figure S5) and two validation cohorts (Supplementary Figure S6). These results also revealed better CSS predictive efficiency for patients with primary RLS.

**Figure 4:**
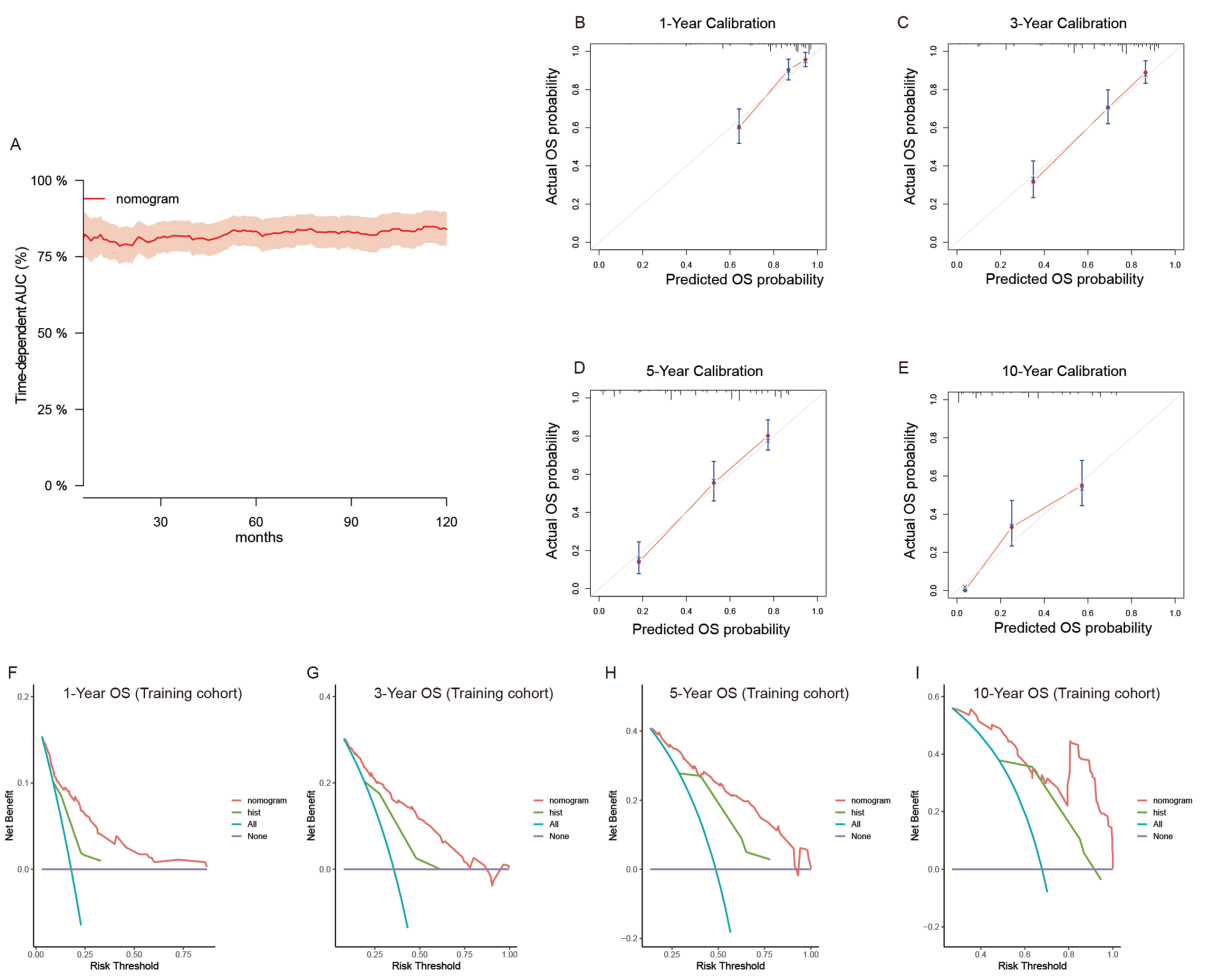
The validation of the OS predictive nomogram for recurrent RLS. **(A)** Time-dependent AUC of using the nomogram to predict overall survival probability within 10 years in the training cohort. The 95% confidence interval was calculated by using the bootstrapping cross-validation method. **(B–E)** Calibration curves of 1-year, 3-year, 5-year and 10-year OS in the training cohort. **(F–I)** Decision curve analysis of the nomogram and pathologic subtypes (hist) for the survival prediction within 10 years.

For recurrent patients, we built independent nomogram models to predict OS and CSS. In the OS predictive model, most patients had total risk points ranging from 64 to 154. The time-dependent AUC was also greater than 0.75 for the prediction of OS within 10 years in the training cohort (Figure 4A). The time-dependent AUCs in the internal (Supplementary Figure S7A) and external (Supplementary Figure S7B) validation cohorts were also greater than 0.75 for most years. Owing to the limited number of cases, we could not fully validate the survival status within 10 years in external cohorts. However, the calibration curves of the model illustrated good consistency between the predicted and observed OS probabilities in both the training cohort (Figures 4B–E) and the validation cohorts (Supplementary Figures S7C–I). Similarly, the clinical benefits of the nomogram model were also shown by DCA curves (Supplementary Figures S7J–P). The model added more net benefits for almost all threshold probabilities in both the training (Figures 4F–I) and two validation cohorts (Supplementary Figures S7C–I). In addition, we further validated the discrimination, consistency and clinical benefits of the CSS nomogram model in the training cohort (Supplementary Figure S8) and validation cohorts (Supplementary Figure S9). The results also revealed better CSS predictive value for patients with recurrent RLS.

### Survival analysis based on risk stratification

**Figure 5:**
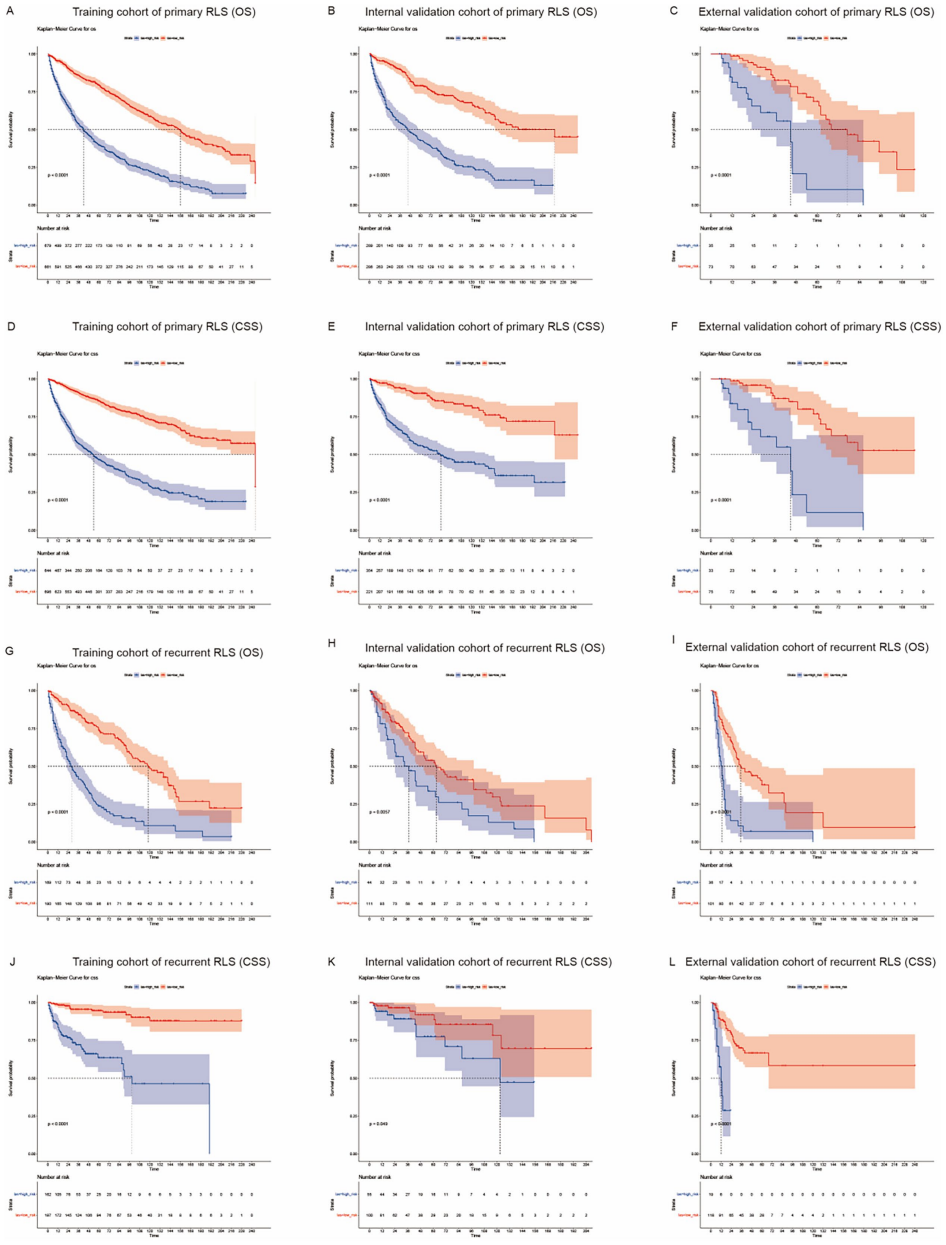
Kaplan-Meier survival curves for RLS patients with different risks stratified by the nomogram. **(A)** OS probability prediction of primary RLS patients in the training cohort at different risks stratified according to the nomogram. **(B–C)** OS probability prediction of primary RLS patients in the validation cohorts at different risks stratified according to the nomogram. **(D)** CSS probability prediction of primary RLS patients in the training cohort at different risks stratified according to the nomogram. **(E–F)** CSS probability prediction of primary RLS patients in the validation cohorts at different risks stratified according to the nomogram. **(G)** OS probability prediction of recurrent RLS patients in the training cohort at different risks stratified according to the nomogram. **(H–I)** OS probability prediction of recurrent RLS patients in the validation cohorts at different risks stratified according to the nomogram. **(J)** CSS probability prediction of recurrent RLS patients in the training cohort at different risks stratified according to the nomogram. **(K–L)** CSS probability prediction of recurrent RLS patients in the validation cohorts at different risks stratified according to the nomogram.

The median survival time was approximately 91 months [interquartile range (IQR): 84–100] and 61 months (IQR: 52–78) for primary patients and recurrent patients, respectively (Figure 1). We used the nomogram model to calculate individuals’ total points and further stratified them according to risk. Patients with RLS were divided into high-risk and low-risk groups. The Kaplan–Meier OS and CSS curves showed great discrimination among the two risk groups in the training set of the primary cohort (*p* < 0.0001). The validation cohort also exhibited good discrimination between the two risk groups (*p* < 0.05) (Figures 5A–F). Similarly, we generated Kaplan–Meier OS and CSS curves for the recurrent cohort (Figures 5G–L). The results revealed good discrimination between the high-risk and low-risk groups. On the basis of the above results, we established highly efficient prognostic models for RLS patients.

## Discussion

RLS is a type of tumor derived from mesenchymal tissue and is the most common type of soft tissue sarcoma (STS) in the retroperitoneal region (16). Patients with RLS are susceptible to recurrence and even death, suggesting a deeper understanding of the pathogenesis of RLS is necessary. Surgical treatment is recognized as an effective treatment for RLS (13). Numerous studies have explored the prognostic factors of RLS, but the sample sizes of those studies were very small, and none included more than a few thousand patients (19). This study contributes valuable insights into the management of primary and recurrent RLS on the basis of large cohort analysis.

In clinical practice and previous reports, we found that there are significant differences in the pathogenesis and prognosis between primary and recurrent RLS, and independent research on the two types of RLS is necessary. The clinical manifestations and surgical procedures for treating primary RLS are relatively simple, and patients with primary RLS tend to have a good prognosis. However, the pathogenesis of recurrent RLS is more complicated, and patients often experience multiple relapses (11, 20). Recurrent RLS is prone to invade adjacent blood vessels and organs, making surgical procedures and combined resection more difficult (21).

Multivariate Cox regression analysis revealed that sex, age, tumor size, pathological subtype, and resection method were significant prognostic factors for primary RLS (Supplementary Table S3). Some results from the Cox regression analysis are consistent with those of previous studies (22, 23). Given the rarity of RLS, we endeavored to enroll 1912 patients with primary RLS from SEER database to construct a preoperative predictive model for those patients. For patients with primary RLS, doctors can use our models to predict survival probability through imaging and needle biopsy examinations. Computerized tomography (CT) or ultrasonic testing can be used to evaluate the tumor size, and needle biopsy pathological results can reveal the pathological subtype of the tumor. These examinations in clinical practice provide indicators for the models and can be used to calculate predictive OS and CSS for patients prior to any treatment. This simplified and highly efficient method can provide guidance for doctors and patients.

Recurrent cases of RLS are usually more aggressive and have poorer prognoses (5, 24). Many studies have explored the risk factors for these patients. Resection methods and pathological subtypes are very important prognostic factors for recurrent RLS. Radical resection promotes a good prognosis for patients with recurrent RLS (17). Likewise, pathological subtypes also play important roles in the recurrence of RLS. Sanjay et al. reported that DDL has the potential for locally aggressive and distant recurrence (25). Tumor size is another important predictor for patients with recurrent RLS. In a previous cohort reported by Yi-xi Wu et al., recurrent patients with tumor sizes larger than 20 cm experienced poor survival outcomes (26). James et al. reported that the tumor growth rate and tumor size affect the likelihood of local recurrence (25). The occurrence pattern remains a pivotal indicator of the prognosis of recurrent RLS patients. Local recurrence may influence the prognosis of patients with recurrent RLS. Similarly, distant recurrence has been accepted as a prognostic factor for recurrent RLS (17). However, few high-volume case-based studies have thoroughly explored the predictive value of the occurrence pattern for patients with recurrent RLS.

We used 514 cases of recurrent RLS to build the predictive model and conducted internal and external validations. With respect to the value of occurrence patterns in RLS, few studies have shown the importance of occurrence patterns in the retroperitoneum (27). In the model, we adopted the occurrence pattern to predict the survival status of recurrent patients with RLS. We found that localized recurrence was an important indicator of CSS. This specific feature is different from primary or *de novo* RLS. In terms of the number of recurrent RLS tumors, these patients usually have multifocal tumors (28). In addition, some cases of recurrent RLS are accompanied by a change in pathological differentiation. Carolyn et al. reported that if WDL recurs as DDL, the recurrence status may be impacted (23, 29). These manifestations inevitably influence survival outcomes. Some previous studies have shown that primary RLS has a better prognosis than recurrent RLS does (25, 30). This difference may be explained by the different biological mechanisms of primary and recurrent RLS.

Adjuvant therapies significantly influence the prognosis of RLS patients, notably enhancing cure rates and improving survival outcomes, particularly for those with larger tumors or initially unresectable lesions (7, 23). These treatments can facilitate tumor downstaging or resection margin improvement, potentially rendering the tumor respectable and augmenting both short-term and long-term patient outcomes. Our study, while recognizing the profound impact of chemotherapy, was constrained by extensive data missing, which precluded a comprehensive discussion on the specific efficacies of these treatments. This limitation underscores the need for future studies to collect comprehensive adjuvant therapy data to provide more accurate prognostic insights. Despite this constraint, our development of distinct nomograms for primary and recurrent RLS, each grounded in their unique clinical and pathological characteristics, highlights the significant clinical utility of our findings.

With the advancement of basic and clinical research, increasing attention has been focused on the exploration of mechanisms and targeted therapies for RLS. In the present study, we constructed a prognostic model for RLS based on traditional clinical and pathological variables, without including systemic inflammatory markers such as Neutrophil-to-Lymphocyte Ratio (NLR), Platelet-to-Lymphocyte Ratio (PLR), Lymphocyte-to-Monocyte Ratio (LMR), and Systemic Immuno-Inflammation Index (SII) (31, 32). The prognostic and predictive value of these markers in STS has gained increasing attention. For instance, NLR and PLR, as indicators of inflammatory response, have been associated with the prognosis of various cancers, including STS. The study by Fausti et al. demonstrated that LMR could predict progression-free survival in STS patients treated with Trabectedin, particularly in cases of liposarcomas (33). Additionally, circulating monocyte levels have been linked to M2 macrophage infiltration in the tumor microenvironment, suggesting that inflammatory markers might influence treatment response (34). Incorporating these markers into our prognostic model could enhance its predictive power and provide more comprehensive prognostic information for patients with RLS. Future research should consider collecting data on these markers to evaluate their potential contribution to prognostic models. By combining traditional clinical and pathological variables with emerging markers, we can anticipate developing more accurate and comprehensive prognostic models that provide stronger support for personalized treatment strategies for patients with RLS.

Our study’s limitations also include its retrospective nature, reliance on institutional medical records and the SEER database, lack of prospective data, missing important therapeutic indicators like adjuvant therapies, absence of tumor necrosis and mitotic count for accurate pathological grading, no discussion on the impact of evolving treatment techniques on prognosis due to its long duration, and missing 10-year validations due to external cohort case limitations.

## Conclusion

To address the divergent clinical manifestations and pathogenic mechanisms between primary and recurrent RLS, we have meticulously crafted distinct nomograms by selecting diverse indicators for each patient subset. These large cohort-based models, distinguished by their high predictive accuracy, substantial clinical utility, and precise prognostic capabilities, will provide indispensable decisional support to both clinicians and patients.

## Data Availability

Data analyzed in the study are available upon request pending application and authority approval. Requests to access the datasets should be directed to Yisheng Pan, BDYYpanyisheng@163.com.

## Footnotes

^1^ https://seer.cancer.gov/datasoftware/
