# Supplementary material for: The independent prognostic nomogram models for primary and recurrent retroperitoneal liposarcoma: a population-based cohort study
Source: Front Med (Lausanne). 2025 Aug 6;12:1642820. doi: 10.3389/fmed.2025.1642820 (PMC12364930; doi:10.3389/fmed.2025.1642820)
Supplement: Supplementary file 1 [file Supplementary_file_1.docx]

**Supplementary Table 1**. Comparison of clinical and pathological characteristics between training and validation sets of all primary patients.

| **Variable** | **Training cohort, N = 1,340** | **Validation cohort, N = 575** | **P-value** |
| --- | --- | --- | --- |
| **Sex** |  |  | 0.33 |
| Female | 597 (45%) | 270 (47%) |  |
| Male | 743 (55%) | 305 (53%) |  |
| **Age** | 62 (53 – 71) | 63 (53 – 72) | 0.48 |
| **Income** |  |  | 0.47 |
| High | 616 (46%) | 273 (47%) |  |
| Middle | 486 (36%) | 213 (37%) |  |
| Low | 238 (18%) | 89 (15%) |  |
| **City** |  |  | 0.65 |
| Metropolitan | 1,207 (90%) | 514 (89%) |  |
| Nonmetropolitan | 133 (9.9%) | 61 (11%) |  |
| **Tumor size** | 200 (130 – 280) | 200 (125 – 290) | 0.79 |
| **T** |  |  | 0.19 |
| T1 | 76 (5.7%) | 39 (6.8%) |  |
| T2 | 140 (10%) | 70 (12%) |  |
| T3 | 247 (18%) | 86 (15%) |  |
| T4 | 877 (65%) | 380 (66%) |  |
| **N** |  |  | 0.68 |
| N0 | 1,053 (79%) | 459 (80%) |  |
| N1 | 28 (2.1%) | 9 (1.6%) |  |
| Unknown | 259 (19%) | 107 (19%) |  |
| **M** |  |  | 0.83 |
| M0 | 1,013 (76%) | 439 (76%) |  |
| M1 | 70 (5.2%) | 32 (5.6%) |  |
| Unknown | 257 (19%) | 104 (18%) |  |
| **TNM stage** |  |  | 0.92 |
| Stage1（I-II） | 633 (47%) | 277 (48%) |  |
| Stage2（III-IV） | 414 (31%) | 173 (30%) |  |
| Unknown | 293 (22%) | 125 (22%) |  |
| **Grade** |  |  | 0.21 |
| Well differentiated | 589 (44%) | 251 (44%) |  |
| Moderately differentiated | 113 (8.4%) | 38 (6.6%) |  |
| Poorly differentiated | 175 (13%) | 81 (14%) |  |
| Undifferentiated | 238 (18%) | 89 (15%) |  |
| Unknown | 225 (17%) | 116 (20%) |  |
| **Chemotherapy** |  |  | 0.88 |
| No/Unknown | 1,164 (87%) | 498 (87%) |  |
| Yes | 176 (13%) | 77 (13%) |  |
| **Pathological subtypes** |  |  | 0.064 |
| WDL | 540 (40%) | 233 (41%) |  |
| MLS | 81 (6.0%) | 32 (5.6%) |  |
| PLS | 31 (2.3%) | 18 (3.1%) |  |
| DDL | 593 (44%) | 231 (40%) |  |
| Liposarcoma, NOS | 95 (7.1%) | 61 (11%) |  |
| **Surgery** |  |  | 0.96 |
| No surgery | 141 (11%) | 59 (10%) |  |
| Partial surgical | 523 (39%) | 222 (39%) |  |
| Total surgical | 676 (50%) | 294 (51%) |  |
| **Overall survival months** | 45 (16 – 93) | 42 (16 – 93) | 0.66 |

WDL Well-differentiated liposarcoma, MLS Myxoid cell liposarcoma, PLS Pleomorphic liposarcoma, DDL Dedifferentiated liposarcoma. Categorical data are expressed as frequencies (percentages) and continuous variables are expressed as the median (Q1-Q3), Bold P value refers to P < 0.05

**Supplementary Table 2**. Comparison of clinical and pathological characteristics between training and validation sets of all recurrent patients.

| **Variable** | **Training cohort, N = 359** | **Validation cohort, N = 155** | **P-value** |
| --- | --- | --- | --- |
| **Sex** |  |  | 0.3 |
| Female | 127 (35%) | 62 (40%) |  |
| Male | 232 (65%) | 93 (60%) |  |
| **Age** | 68 (60 – 76) | 68 (57 – 76) | 0.2 |
| **Income** |  |  | 0.3 |
| High | 170 (47%) | 83 (54%) |  |
| Middle | 124 (35%) | 51 (33%) |  |
| Low | 65 (18%) | 21 (14%) |  |
| **City** |  |  | 0.6 |
| Metropolitan | 313 (87%) | 138 (89%) |  |
| Nonmetropolitan | 46 (13%) | 17 (11%) |  |
| **Tumor size** | 162 (90, 230) | 151 (90, 250) | >0.9 |
| **T** |  |  | 0.6 |
| T1 | 37 (10%) | 14 (9.0%) |  |
| T2 | 68 (19%) | 37 (24%) |  |
| T3 | 62 (17%) | 26 (17%) |  |
| T4 | 192 (53%) | 78 (50%) |  |
| **N** |  |  | 0.5 |
| N0 | 292 (81%) | 119 (77%) |  |
| N1 | 7 (1.9%) | 3 (1.9%) |  |
| Unknown | 60 (17%) | 33 (21%) |  |
| **M** |  |  | 0.3 |
| M0 | 290 (81%) | 120 (77%) |  |
| M1 | 13 (3.6%) | 3 (1.9%) |  |
| Unknown | 56 (16%) | 32 (21%) |  |
| **TNM stage** |  |  | 0.4 |
| Stage1（I-II） | 190 (53%) | 75 (48%) |  |
| Stage2（III-IV） | 104 (29%) | 44 (28%) |  |
| Unknown | 65 (18%) | 36 (23%) |  |
| **Grade** |  |  | 0.9 |
| Well differentiated | 144 (40%) | 66 (43%) |  |
| Moderately differentiated | 23 (6.4%) | 13 (8.4%) |  |
| Poorly differentiated | 56 (16%) | 21 (14%) |  |
| Undifferentiated | 63 (18%) | 25 (16%) |  |
| Unknown | 73 (20%) | 30 (19%) |  |
| **Chemotherapy** |  |  | 0.13 |
| No/Unknown | 332 (92%) | 137 (88%) |  |
| Yes | 27 (7.5%) | 18 (12%) |  |
| **Pathological subtypes** |  |  | 0.068 |
| WDL | 138 (38%) | 59 (38%) |  |
| MLS | 12 (3.3%) | 14 (9.0%) |  |
| PLS | 6 (1.7%) | 3 (1.9%) |  |
| DDL | 171 (48%) | 62 (40%) |  |
| Liposarcoma, NOS | 32 (8.9%) | 17 (11%) |  |
| **Surgery** |  |  | 0.9 |
| No surgery | 44 (12%) | 17 (11%) |  |
| Partial surgical | 142 (40%) | 64 (41%) |  |
| Total surgical | 173 (48%) | 74 (48%) |  |
| Occurrence pattern |  |  | 0.46 |
| Localized | 193 (54%) | 86 (55%) |  |
| Non-localized | 157 (44%) | 68 (44%) |  |
| Unknown | 9 (2.5%) | 1 (0.6%) |  |
| **Overall survival months** | 34 (12 – 78) | 32 (15 – 66) | 0.7 |

WDL Well-differentiated liposarcoma, MLS Myxoid cell liposarcoma, PLS Pleomorphic liposarcoma, DDL Dedifferentiated liposarcoma. Categorical data are expressed as frequencies (percentages) and continuous variables are expressed as the median (Q1-Q3), Bold P value refers to P < 0.05.

**Supplementary Table 3.** Univariate and multivariate analysis of clinicopathologic variables associated with OS in primary patients.

|  |  | **Univariate analysis** | |  | **Multivariate analysis** | |  |
| --- | --- | --- | --- | --- | --- | --- | --- |
| **Variable** | **N** | **HR** | **95% CI** | **P value** | **HR** | **95% CI** | **P value** |
| **Sex** |  |  |  | **<0.001** |  |  | **0.005** |
| Female | 597 | — | — |  | — | — |  |
| Male | 743 | 1.47 | 1.26-1.71 |  | 1.25 | 1.07-1.46 |  |
| **Age** |  |  |  | **<0.001** |  |  | **<0.001** |
| Young | 567 | — | — |  | — | — |  |
| Middle | 533 | 1.62 | 1.36-1.94 |  | 1.43 | 1.20-1.71 |  |
| Old | 240 | 3.14 | 2.57-3.85 |  | 2.58 | 2.08-3.20 |  |
| **Income** |  |  |  | 0.066 |  |  |  |
| High | 616 | — | — |  |  |  |  |
| Middle | 486 | 1.06 | 0.90-1.26 |  |  |  |  |
| Low | 238 | 1.28 | 1.04-1.57 |  |  |  |  |
| **City** |  |  |  | **0.034** |  |  | 0.083 |
| Metropolitan | 1,207 | — | — |  | — | — |  |
| Nonmetropolitan | 133 | 1.30 | 1.03-1.64 |  | 1.24 | 0.98-1.57 |  |
| **Tumor size** | 1,340 | 1.00 | 1.00-1.00 | **0.002** | 1.00 | 1.00-1.00 | **<0.001** |
| **T** |  |  |  | 0.18 |  |  |  |
| T1 | 76 | — | — |  |  |  |  |
| T2 | 140 | 0.92 | 0.60-1.41 |  |  |  |  |
| T3 | 247 | 1.17 | 0.80-1.71 |  |  |  |  |
| T4 | 877 | 1.21 | 0.86-1.72 |  |  |  |  |
| **N** |  |  |  | **0.007** |  |  |  |
| N0 | 1,053 | — | — |  |  |  |  |
| N1 | 28 | 2.27 | 1.42-3.63 |  |  |  |  |
| Unknown | 259 | 0.94 | 0.78-1.14 |  |  |  |  |
| **M** |  |  |  | **<0.001** |  |  |  |
| M0 | 1,013 | — | — |  |  |  |  |
| M1 | 70 | 4.94 | 3.73-6.53 |  |  |  |  |
| Unknown | 257 | 1.00 | 0.83-1.21 |  |  |  |  |
| **TNM stage** |  |  |  | **<0.001** |  |  | **<0.001** |
| Stage1（I-II） | 633 | — | — |  | — | — |  |
| Stage2（III-IV） | 414 | 2.55 | 2.14-3.04 |  | 1.60 | 1.30-1.97 |  |
| Unknown | 293 | 1.30 | 1.07-1.59 |  | 1.01 | 0.82-1.24 |  |
| **Grade** |  |  |  | **<0.001** |  |  |  |
| Well differentiated | 589 | — | — |  |  |  |  |
| Moderately differentiated | 113 | 1.32 | 0.99-1.75 |  |  |  |  |
| Poorly differentiated | 175 | 2.67 | 2.14-3.32 |  |  |  |  |
| Undifferentiated | 238 | 2.55 | 2.09-3.11 |  |  |  |  |
| Unknown | 225 | 1.95 | 1.52-2.49 |  |  |  |  |
| **Chemotherapy** |  |  |  | **<0.001** |  |  |  |
| No/Unknown | 1,164 | — | — |  |  |  |  |
| Yes | 176 | 2.64 | 2.16-3.24 |  |  |  |  |
| **Pathological subtypes** |  |  |  | **<0.001** |  |  | **<0.001** |
| WDL | 540 | — | — |  | — | — |  |
| MLS | 81 | 1.73 | 1.28-2.33 |  | 1.74 | 1.28-2.37 |  |
| PLS | 31 | 2.02 | 1.27-3.23 |  | 1.75 | 1.08-2.83 |  |
| DDL | 593 | 2.51 | 2.11-2.98 |  | 2.11 | 1.71-2.61 |  |
| Liposarcoma, NOS | 95 | 1.90 | 1.40-2.59 |  | 0.98 | 0.71-1.37 |  |
| **Surgery** |  |  |  | **<0.001** |  |  | **<0.001** |
| No surgery | 141 | — | — |  | — | — |  |
| Partial surgical | 523 | 0.25 | 0.20-0.32 |  | 0.29 | 0.23-0.37 |  |
| Total surgical | 676 | 0.26 | 0.21-0.32 |  | 0.23 | 0.18-0.30 |  |

OS Overall survival;  HR Hazard ratio, CI Confidence interval, WDL Well-differentiated liposarcoma, MLS Myxoid cell liposarcoma, PLS Pleomorphic liposarcoma, DDL Dedifferentiated liposarcoma. The cutoff value was the median value of variable. Bold P value refers to P < 0.05.

**Supplementary Table 4**. Univariate and multivariate analysis of clinicopathologic variables associated with CSS in primary patients.

|  |  | **Univariate analysis** | |  | **Multivariate analysis** | |  |
| --- | --- | --- | --- | --- | --- | --- | --- |
| **Variable** | **N** | **HR** | **95% CI** | **P value** | **HR** | **95% CI** | **P value** |
| **Sex** |  |  |  | **<0.001** |  |  | 0.052 |
| Female | 597 | — | — |  | — | — |  |
| Male | 743 | 1.45 | 1.21-1.74 |  | 1.20 | 1.00-1.44 |  |
| **Age** |  |  |  | **<0.001** |  |  | **<0.001** |
| Young | 567 | — | — |  | — | — |  |
| Middle | 533 | 1.49 | 1.22-1.82 |  | 1.28 | 1.04-1.57 |  |
| Old | 240 | 2.22 | 1.73-2.85 |  | 1.82 | 1.40-2.36 |  |
| **Income** |  |  |  | 0.31 |  |  |  |
| High | 616 | — | — |  |  |  |  |
| Middle | 486 | 0.96 | 0.79-1.18 |  |  |  |  |
| Low | 238 | 1.17 | 0.92-1.49 |  |  |  |  |
| **City** |  |  |  | 0.42 |  |  |  |
| Metropolitan | 1,207 | — | — |  |  |  |  |
| Nonmetropolitan | 133 | 1.13 | 0.84-1.51 |  |  |  |  |
| **Tumor size** | 1,340 | 1.00 | 1.00-1.00 | **<0.001** | 1.00 | 1.00-1.00 | **<0.001** |
| **T** |  |  |  | **0.007** |  |  |  |
| T1 | 76 | — | — |  |  |  |  |
| T2 | 140 | 1.08 | 0.61-1.90 |  |  |  |  |
| T3 | 247 | 1.44 | 0.87-2.40 |  |  |  |  |
| T4 | 877 | 1.69 | 1.05-2.71 |  |  |  |  |
| **N** |  |  |  | **<0.001** |  |  |  |
| N0 | 1,053 | — | — |  |  |  |  |
| N1 | 28 | 2.92 | 1.80-4.76 |  |  |  |  |
| Unknown | 259 | 0.96 | 0.76-1.20 |  |  |  |  |
| **M** |  |  |  | **<0.001** |  |  |  |
| M0 | 1,013 | — | — |  |  |  |  |
| M1 | 70 | 6.03 | 4.47-8.14 |  |  |  |  |
| Unknown | 257 | 1.05 | 0.83-1.32 |  |  |  |  |
| **TNM stage** |  |  |  | **<0.001** |  |  | **<0.001** |
| Stage1（I-II） | 633 | — | — |  | — | — |  |
| Stage2（III-IV） | 414 | 3.53 | 2.86-4.36 |  | 1.90 | 1.49-2.42 |  |
| Unknown | 293 | 1.60 | 1.26-2.05 |  | 1.18 | 0.91-1.52 |  |
| **Grade** |  |  |  | **<0.001** |  |  |  |
| Well differentiated | 589 | — | — |  |  |  |  |
| Moderately differentiated | 113 | 1.97 | 1.41-2.73 |  |  |  |  |
| Poorly differentiated | 175 | 4.09 | 3.17-5.30 |  |  |  |  |
| Undifferentiated | 238 | 3.70 | 2.91-4.71 |  |  |  |  |
| Unknown | 225 | 2.39 | 1.76-3.24 |  |  |  |  |
| **Chemotherapy** |  |  |  | **<0.001** |  |  |  |
| No/Unknown | 1,164 | — | — |  |  |  |  |
| Yes | 176 | 3.43 | 2.75-4.27 |  |  |  |  |
| **Pathological subtypes** |  |  |  | **<0.001** |  |  | **<0.001** |
| WDL | 540 | — | — |  | — | — |  |
| MLS | 81 | 3.02 | 2.14-4.27 |  | 2.82 | 1.98-4.00 |  |
| PLS | 31 | 3.40 | 2.01-5.74 |  | 2.56 | 1.49-4.39 |  |
| DDL | 593 | 3.99 | 3.19-5.00 |  | 3.05 | 2.34-3.97 |  |
| Liposarcoma, NOS | 95 | 2.31 | 1.54-3.46 |  | 1.13 | 0.74-1.74 |  |
| **Surgery** |  |  |  | **<0.001** |  |  | **<0.001** |
| No surgery | 141 | — | — |  | — | — |  |
| Partial surgical | 523 | 0.24 | 0.18-0.31 |  | 0.25 | 0.18-0.33 |  |
| Total surgical | 676 | 0.27 | 0.21-0.35 |  | 0.20 | 0.15-0.27 |  |

CSS Cancer-specific survival; HR Hazard ratio, CI Confidence interval, WDL Well-differentiated liposarcoma, MLS Myxoid cell liposarcoma, PLS Pleomorphic liposarcoma, DDL Dedifferentiated liposarcoma. Bold P value refers to P < 0.05.

**Supplementary Table 5**. Univariate and multivariate analysis of clinicopathologic variables associated with OS in recurrent patients.

|  |  | **Univariate analysis** | |  | **Multivariate analysis** | |  |
| --- | --- | --- | --- | --- | --- | --- | --- |
| **Variable** | **N** | **HR** | **95% CI** | **P value** | **HR** | **95% CI** | **P value** |
| **Sex** |  |  |  | 0.084 |  |  |  |
| Female | 127 | — | — |  |  |  |  |
| Male | 232 | 1.30 | 0.96-1.77 |  |  |  |  |
| **Age** |  |  |  | **<0.001** |  |  | **<0.001** |
| Young | 83 | — | — |  | — | — |  |
| Middle | 161 | 1.37 | 0.91-2.07 |  | 1.38 | 0.90-2.11 |  |
| Old | 115 | 3.08 | 2.05-4.65 |  | 3.27 | 2.14-5.01 |  |
| **Income** |  |  |  | 0.60 |  |  |  |
| High | 170 | — | — |  |  |  |  |
| Middle | 124 | 0.90 | 0.65-1.23 |  |  |  |  |
| Low | 65 | 1.09 | 0.74-1.61 |  |  |  |  |
| **City** |  |  |  | 0.39 |  |  |  |
| Metropolitan | 313 | — | — |  |  |  |  |
| Nonmetropolitan | 46 | 0.83 | 0.53-1.29 |  |  |  |  |
| **Tumor size** | 359 | 1.00 | 1.00-1.00 | 0.60 |  |  |  |
| **T** |  |  |  | 0.66 |  |  |  |
| T1 | 37 | — | — |  |  |  |  |
| T2 | 68 | 1.20 | 0.66-2.19 |  |  |  |  |
| T3 | 62 | 1.28 | 0.71-2.32 |  |  |  |  |
| T4 | 192 | 1.36 | 0.81-2.30 |  |  |  |  |
| **N** |  |  |  | 0.038 |  |  |  |
| N0 | 292 | — | — |  |  |  |  |
| N1 | 7 | 4.14 | 1.68-10.2 |  |  |  |  |
| Unknown | 60 | 0.98 | 0.68-1.42 |  |  |  |  |
| **M** |  |  |  | **<0.001** |  |  |  |
| M0 | 290 | — | — |  |  |  |  |
| M1 | 13 | 4.49 | 2.47-8.16 |  |  |  |  |
| Unknown | 56 | 0.97 | 0.66-1.42 |  |  |  |  |
| **TNM stage** |  |  |  | **<0.001** |  |  | **<0.001** |
| Stage1（I-II） | 190 | — | — |  | — | — |  |
| Stage2（III-IV） | 104 | 2.51 | 1.81-3.48 |  | 2.18 | 1.49-3.20 |  |
| Unknown | 65 | 1.31 | 0.90-1.91 |  | 1.36 | 0.91-2.04 |  |
| **Grade** |  |  |  | **<0.001** |  |  |  |
| Well differentiated | 144 | — | — |  |  |  |  |
| Moderately differentiated | 23 | 1.63 | 0.91-2.93 |  |  |  |  |
| Poorly differentiated | 56 | 3.41 | 2.28-5.09 |  |  |  |  |
| Undifferentiated | 63 | 3.33 | 2.24-4.95 |  |  |  |  |
| Unknown | 73 | 3.52 | 2.24-5.52 |  |  |  |  |
| **Chemotherapy** |  |  |  | 0.028 |  |  |  |
| No/Unknown | 332 | — | — |  |  |  |  |
| Yes | 27 | 1.80 | 1.11-2.94 |  |  |  |  |
| **Pathological subtypes** |  |  |  | **<0.001** |  |  | **<0.001** |
| WDL | 138 | — | — |  | — | — |  |
| MLS | 12 | 1.52 | 0.74-3.09 |  | 1.66 | 0.79-3.49 |  |
| PLS | 6 | 3.08 | 1.11-8.57 |  | 1.72 | 0.60-4.92 |  |
| DDL | 171 | 2.88 | 2.05-4.03 |  | 2.25 | 1.50-3.36 |  |
| Liposarcoma-NOS | 32 | 4.44 | 2.72-7.22 |  | 2.57 | 1.47-4.48 |  |
| **Surgery** |  |  |  | **<0.001** |  |  | **<0.001** |
| No surgery | 44 | — | — |  | — | — |  |
| Partial surgical | 142 | 0.25 | 0.16-0.38 |  | 0.27 | 0.16-0.46 |  |
| Total surgical | 173 | 0.37 | 0.25-0.56 |  | 0.29 | 0.17-0.48 |  |
| **Occurrence pattern** |  |  |  | **0.002** |  |  | 0.2 |
| Localized | 193 | — | — |  | — | — |  |
| Non-localized | 157 | 1.60 | 1.20-2.13 |  | 1.34 | 0.99-1.82 |  |
| Unknown | 9 | 2.26 | 1.05-4.90 |  | 1.11 | 0.48-2.58 |  |

OS Overall survival;  HR Hazard ratio, CI Confidence interval, WDL Well-differentiated liposarcoma, MLS Myxoid cell liposarcoma, PLS Pleomorphic liposarcoma, DDL Dedifferentiated liposarcoma. The cutoff value was the median value of variable. Bold P value refers to P < 0.05

**Supplementary Table 6**. Univariate and multivariate analysis of clinicopathologic variables associated with CSS in recurrent patients.

|  |  | **Univariate analysis** | |  | **Multivariate analysis** | |  |
| --- | --- | --- | --- | --- | --- | --- | --- |
| **Variable** | **N** | **HR** | **95% CI** | **P value** | **HR** | **95% CI** | **P value** |
| **Sex** |  |  |  | 0.75 |  |  |  |
| Female | 127 | — | — |  |  |  |  |
| Male | 232 | 0.92 | 0.54-1.56 |  |  |  |  |
| **Age** |  |  |  | **0.036** |  |  | **0.028** |
| Young | 83 | — | — |  | — | — |  |
| Middle | 161 | 2.05 | 0.94-4.50 |  | 2.38 | 1.05-5.41 |  |
| Old | 115 | 2.75 | 1.21-6.27 |  | 2.90 | 1.22-6.88 |  |
| **Income** |  |  |  | 0.13 |  |  |  |
| High | 170 | — | — |  |  |  |  |
| Middle | 124 | 0.67 | 0.38-1.18 |  |  |  |  |
| Low | 65 | 0.47 | 0.20-1.12 |  |  |  |  |
| **City** |  |  |  | 0.24 |  |  |  |
| Metropolitan | 313 | — | — |  |  |  |  |
| Nonmetropolitan | 46 | 0.6 | 0.24-1.49 |  |  |  |  |
| **Tumor size** | 359 | 1 | 1.00-1.00 | 0.16 |  |  |  |
| **T** |  |  |  | 0.26 |  |  |  |
| T1 | 37 | — | — |  |  |  |  |
| T2 | 68 | 1.49 | 0.39-5.61 |  |  |  |  |
| T3 | 62 | 1.96 | 0.54-7.14 |  |  |  |  |
| T4 | 192 | 2.45 | 0.75-7.94 |  |  |  |  |
| **N** |  |  |  | 0.25 |  |  |  |
| N0 | 292 | — | — |  |  |  |  |
| N1 | 7 | 4.34 | 1.04-18.1 |  |  |  |  |
| Unknown | 60 | 1.1 | 0.56-2.13 |  |  |  |  |
| **M** |  |  |  | **<0.001** |  |  |  |
| M0 | 290 | — | — |  |  |  |  |
| M1 | 13 | 9.38 | 4.32-20.4 |  |  |  |  |
| Unknown | 56 | 1.34 | 0.68-2.63 |  |  |  |  |
| **TNM stage** |  |  |  | **0.007** |  |  | 0.20 |
| Stage1（I-II） | 190 | — | — |  | — | — |  |
| Stage2（III-IV） | 104 | 2.6 | 1.45-4.66 |  | 1.84 | 0.96-3.52 |  |
| Unknown | 65 | 1.35 | 0.66-2.78 |  | 1.47 | 0.69-3.16 |  |
| **Grade** |  |  |  | **<0.001** |  |  |  |
| Well differentiated | 144 | — | — |  |  |  |  |
| Moderately differentiated | 23 | 4.06 | 1.47-11.2 |  |  |  |  |
| Poorly differentiated | 56 | 5.56 | 2.44-12.7 |  |  |  |  |
| Undifferentiated | 63 | 7.12 | 3.28-15.4 |  |  |  |  |
| Unknown | 73 | 3.53 | 1.36-9.19 |  |  |  |  |
| **Chemotherapy** |  |  |  | **0.002** |  |  |  |
| No/Unknown | 332 | — | — |  |  |  |  |
| Yes | 27 | 3.44 | 1.73-6.83 |  |  |  |  |
| **Pathological subtypes** |  |  |  | **<0.001** |  |  | **<0.001** |
| WDL | 138 | — | — |  | — | — |  |
| MLS | 12 | 6.57 | 2.11-20.5 |  | 7.46 | 2.30-24.2 |  |
| PLS | 6 | 9.17 | 1.92-43.9 |  | 4.34 | 0.85-22.2 |  |
| DDL | 171 | 5.87 | 2.67-12.9 |  | 4.63 | 1.95-11.0 |  |
| Liposarcoma-NOS | 32 | 9.65 | 3.66-25.4 |  | 5.98 | 2.07-17.3 |  |
| **Surgery** |  |  |  | **0.002** |  |  | **0.006** |
| No surgery | 44 | — | — |  | — | — |  |
| Partial surgical | 142 | 0.25 | 0.12-0.51 |  | 0.25 | 0.10-0.62 |  |
| Total surgical | 173 | 0.31 | 0.16-0.62 |  | 0.21 | 0.09-0.51 |  |
| **Occurrence pattern** |  |  |  | **0.002** |  |  | **0.017** |
| Localized | 193 | — | — |  | — | — |  |
| Non-localized | 157 | 2.6 | 1.49-4.52 |  | 2.30 | 1.28-4.14 |  |
| Unknown | 9 | 2.86 | 0.66-12.3 |  | 1.33 | 0.28-6.37 |  |

CSS Cancer-specific survival; HR Hazard ratio, CI Confidence interval, WDL Well-differentiated liposarcoma, MLS Myxoid cell liposarcoma, PLS Pleomorphic liposarcoma, DDL Dedifferentiated liposarcoma. Bold P value refers to P < 0.05

**Supplementary Figures**


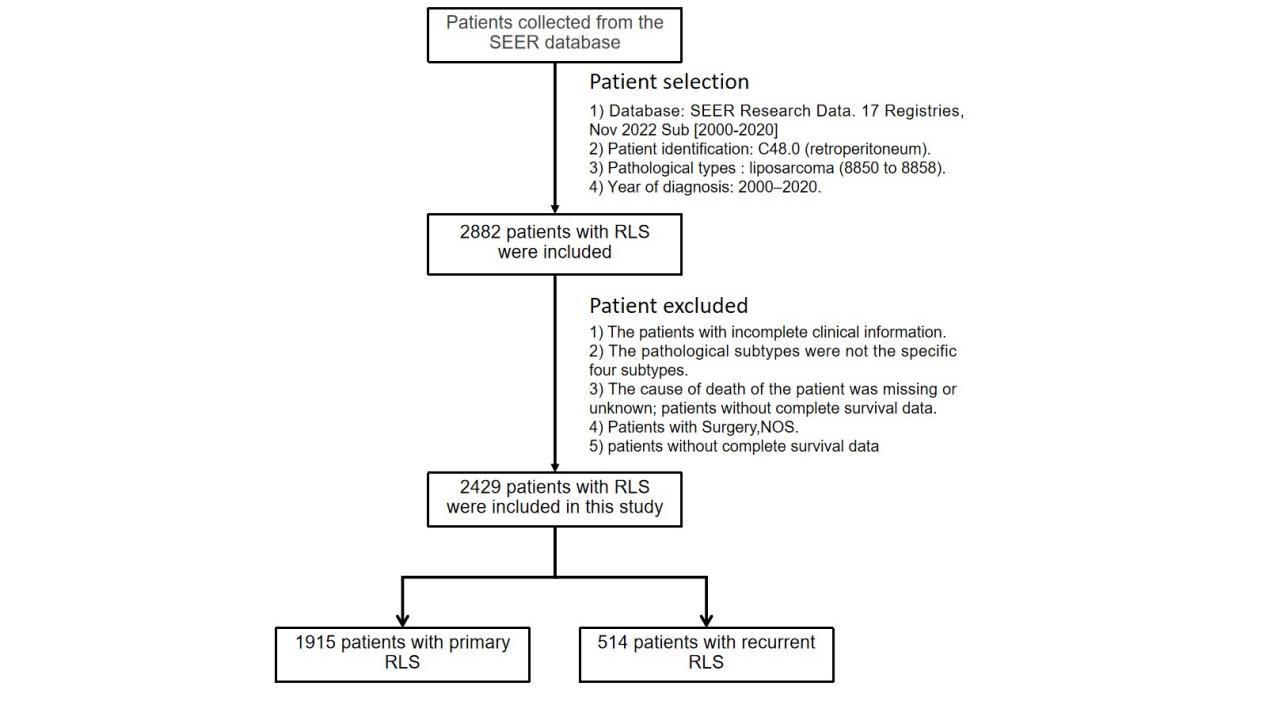


**Supplementary Figure 1**. Enrollment flow diagram of patients with RLS. RLS: retroperitoneal liposarcoma.


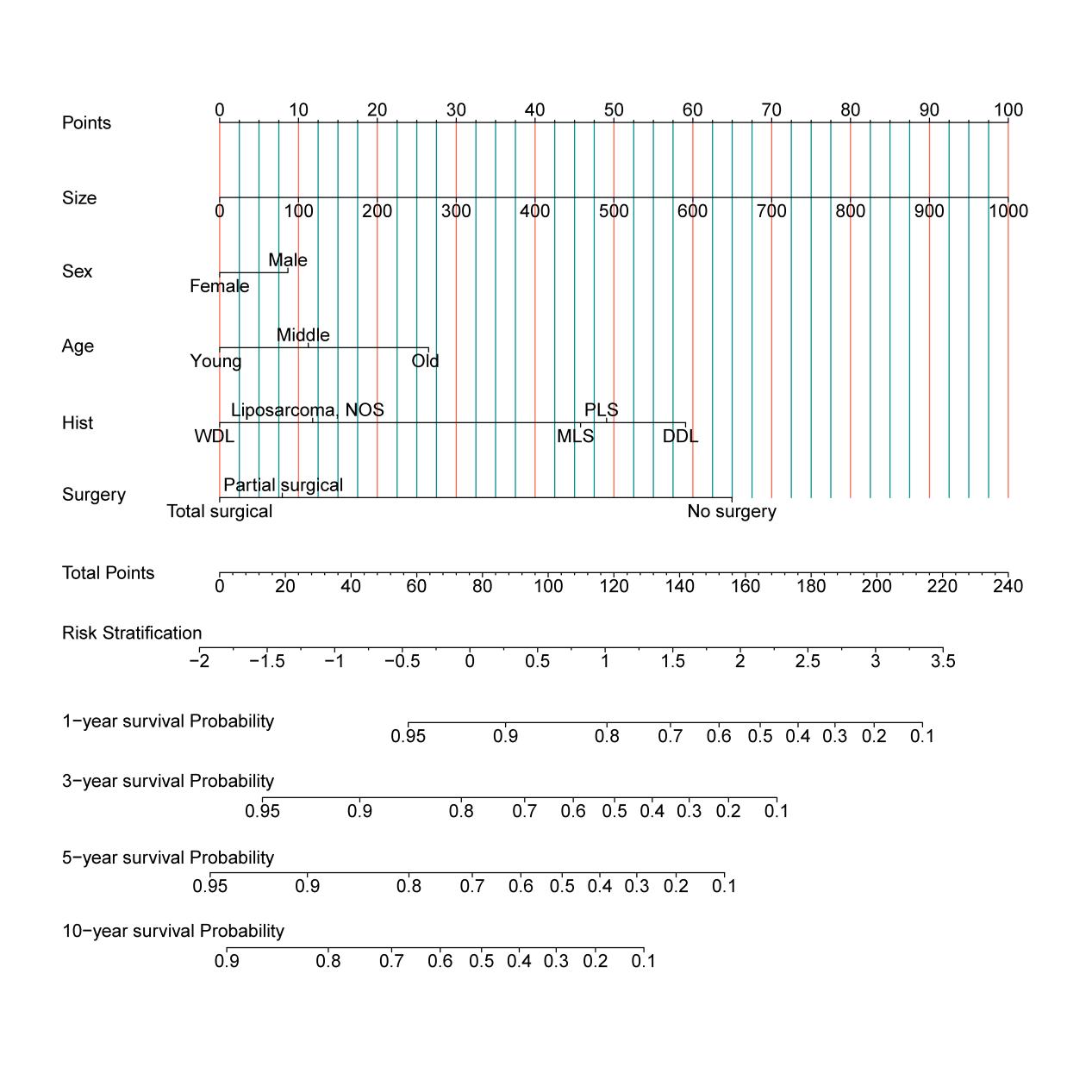


**Supplementary Figure 2**. The nomogram model was built to predict the cancer-specific survival (CSS) status of patients with primary RLS. CSS: cancer-specific survival; RLS, retroperitoneal liposarcoma; WDL, well-differentiated liposarcoma; DDL, dedifferentiated liposarcoma; MLS, myxoid cell liposarcoma; PLS, pleomorphic liposarcoma; liposarcoma NOS: Unable to determine the specific subtype; “Hist”: pathological subtypes; Age, Young: ≦ 60 years old, Middle: 60-75 years old, Old: ≧ 75 years old (Classification criteria: according to the distribution features and previous studies).


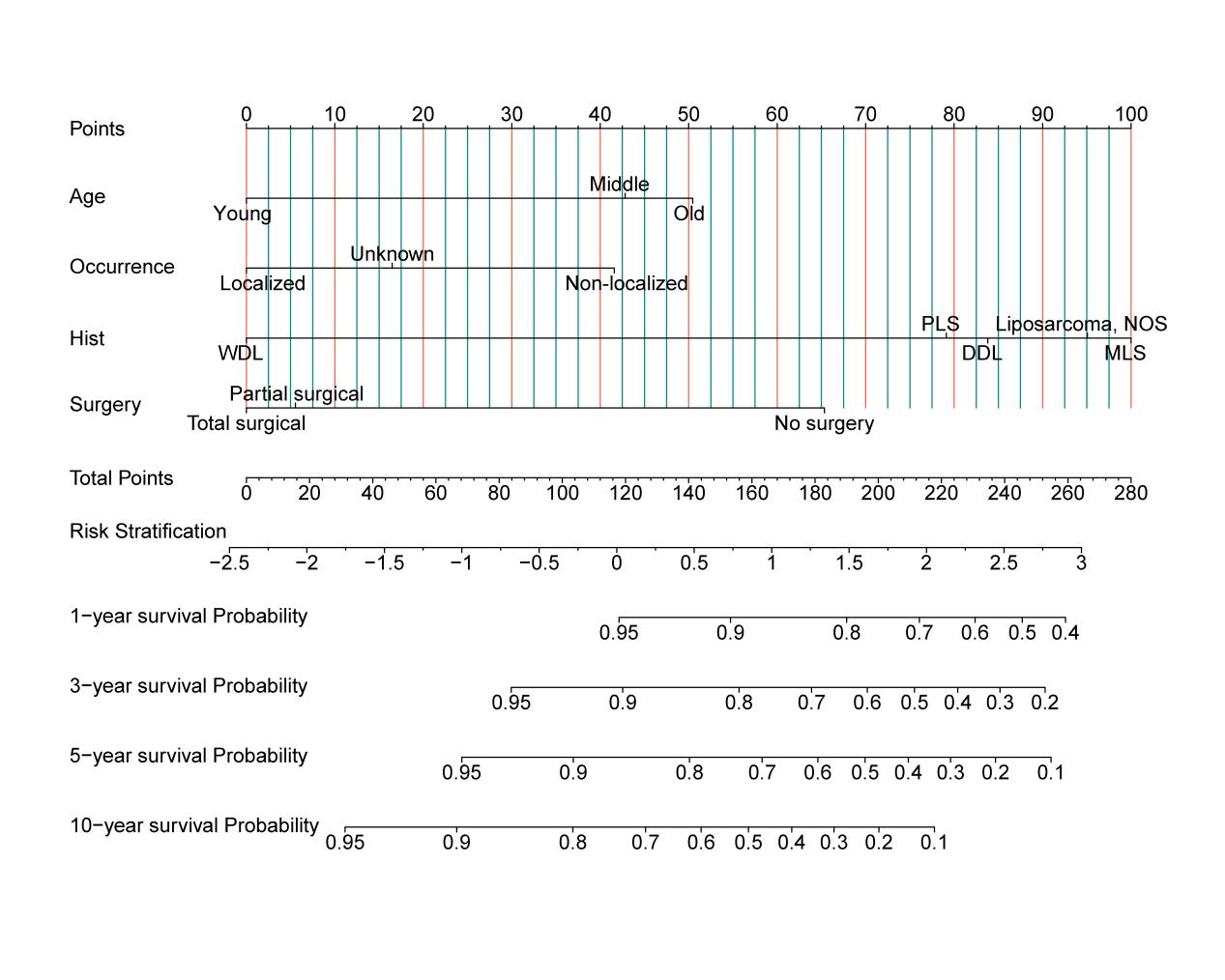


**Supplementary Figure 3**. The nomogram model was built to predict the cancer-specific survival (CSS) status of patients with recurrent RLS. RLS, retroperitoneal liposarcoma; WDL, well-differentiated liposarcoma; DDL, dedifferentiated liposarcoma; MLS, myxoid cell liposarcoma; PLS, pleomorphic liposarcoma; liposarcoma NOS: unable to determine the specific subtype; “Hist”: pathological subtypes; Occurrence pattern (SEER stage) represents the extent of tumor invasion in retroperitoneum; unknown: unable to determine the specific stage; Age, Young: ≦ 60 years old, Middle: 60-75 years old, Old: ≧ 75 years old (Classification criteria: according to the distribution features and previous studies).


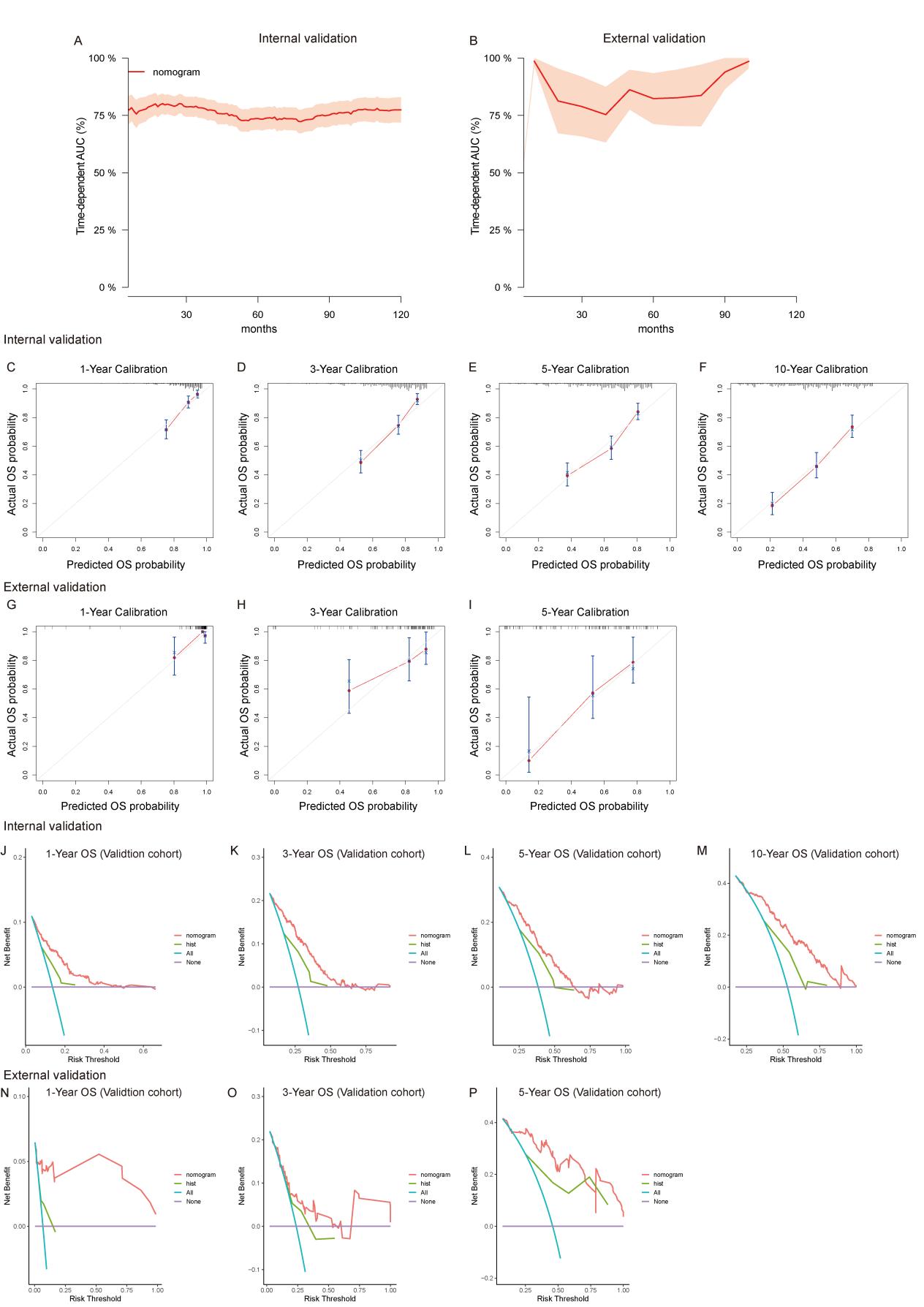


**Supplementary Figure 4**. The internal and external validation of the OS predictive nomogram for primary RLS. (A) Time-dependent AUC of using the nomogram to predict overall survival probability within 10 years in internal validation cohort. The 95% confidence interval was calculated by using the bootstrapping cross-validation method. (B) Time-dependent AUC of using the nomogram to predict overall survival probability within 10 years in external validation cohort. The 95% confidence interval was calculated by using the bootstrapping cross-validation method. (C-F) Calibration curves of 1-year, 3-year, 5-year and 10-year OS in internal validation cohort. (G-I) Calibration curves of 1-year, 3-year and 5-year OS in the external validation cohort. (J-M) Decision curve analysis of the nomogram and pathologic subtypes (hist) for the survival prediction within 10 years in internal validation cohort. (J-M) Decision curve analysis of the nomogram and pathologic subtypes (hist) for the survival prediction within 5 years in the external validation cohort.


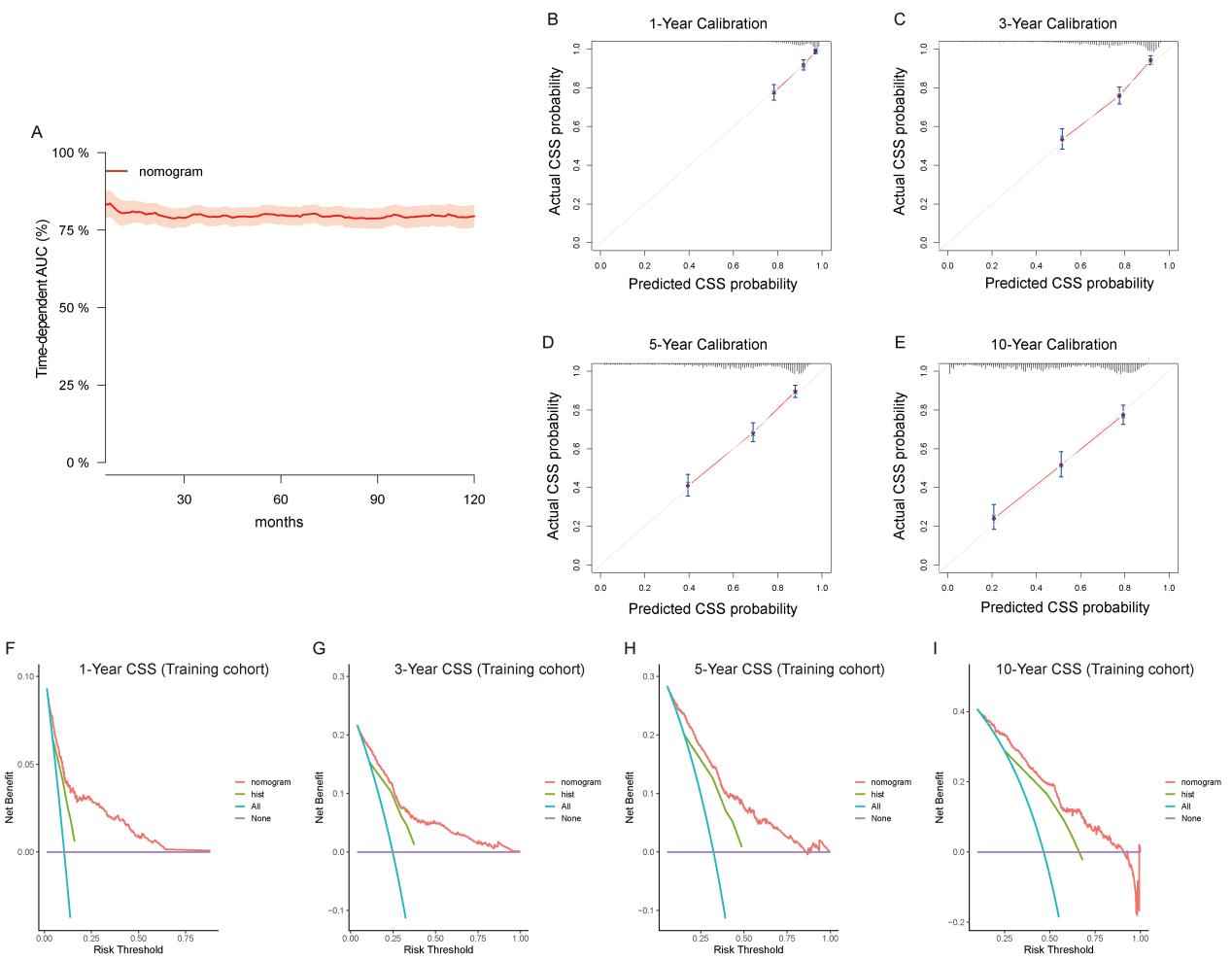


**Supplementary Figure 5**. The validation of the CSS predictive nomogram for primary RLS in the training cohort. (A) Time-dependent AUC of using the nomogram to predict CSS probability within 10 years in the training cohort. The 95% confidence interval was calculated by using the bootstrapping cross-validation method. (B-E) Calibration curves of 1-year, 3-year, 5-year and 10-year CSS in the training cohort. (F-I) Decision curve analysis of the nomogram and pathologic subtypes (hist) for the survival prediction within 10 years.


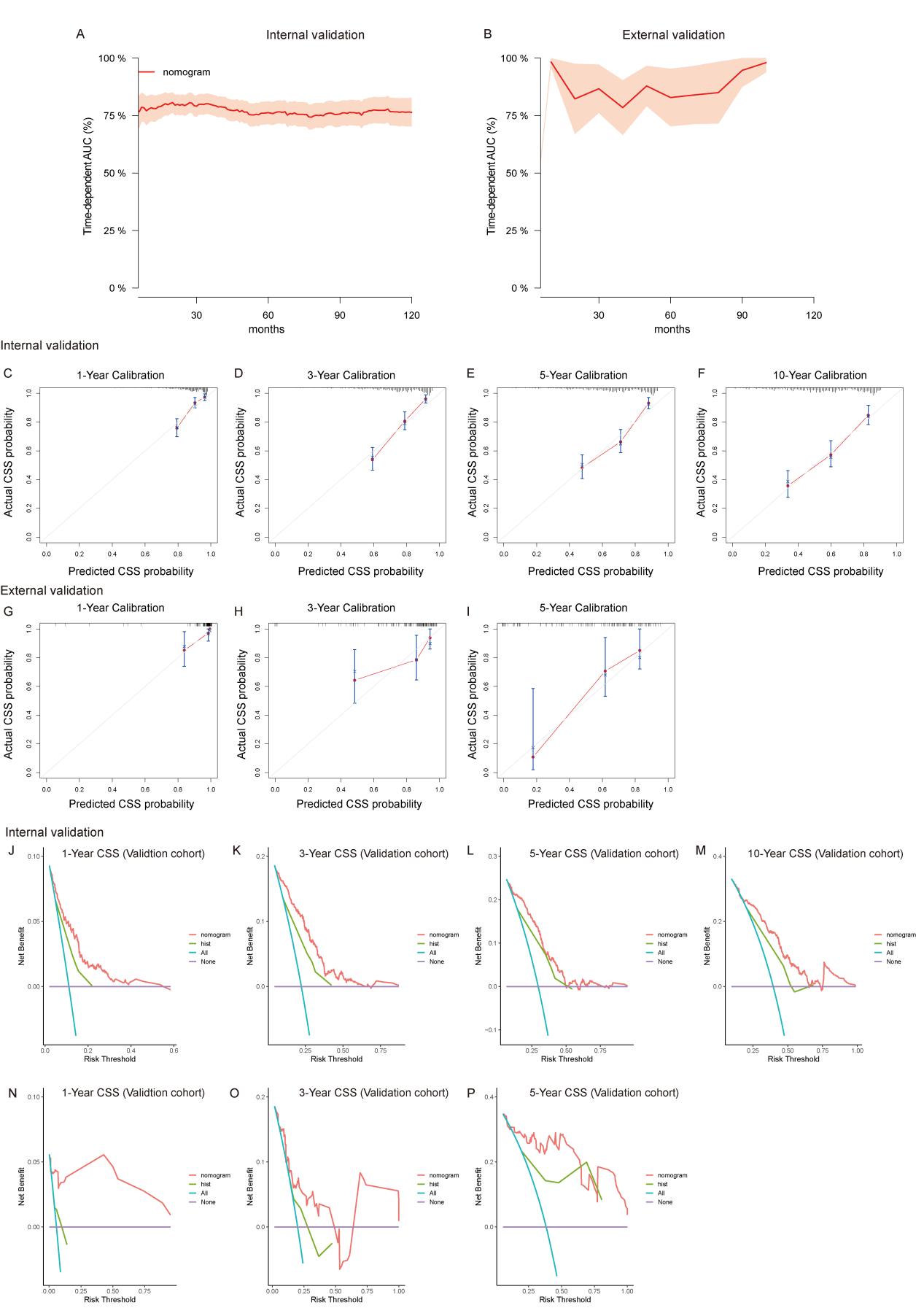


**Supplementary Figure 6**. The internal and external validation of the CSS predictive nomogram for primary RLS. (A) Time-dependent AUC of using the nomogram to predict CSS probability within 10 years in internal validation cohort. The 95% confidence interval was calculated by using the bootstrapping cross-validation method. (B) Time-dependent AUC of using the nomogram to predict CSS probability within 10 years in external validation cohort. The 95% confidence interval was calculated by using the bootstrapping cross-validation method. (C-F) Calibration curves of 1-year, 3-year, 5-year and 10-year CSS in internal validation cohort. (G-I) Calibration curves of 1-year, 3-year and 5-year CSS in the external validation cohort. (J-M) Decision curve analysis of the nomogram and pathologic subtypes (hist) for the survival prediction within 10 years in internal validation cohort. (J-M) Decision curve analysis of the nomogram and pathologic subtypes (hist) for the survival prediction within 5 years in the external validation cohort.


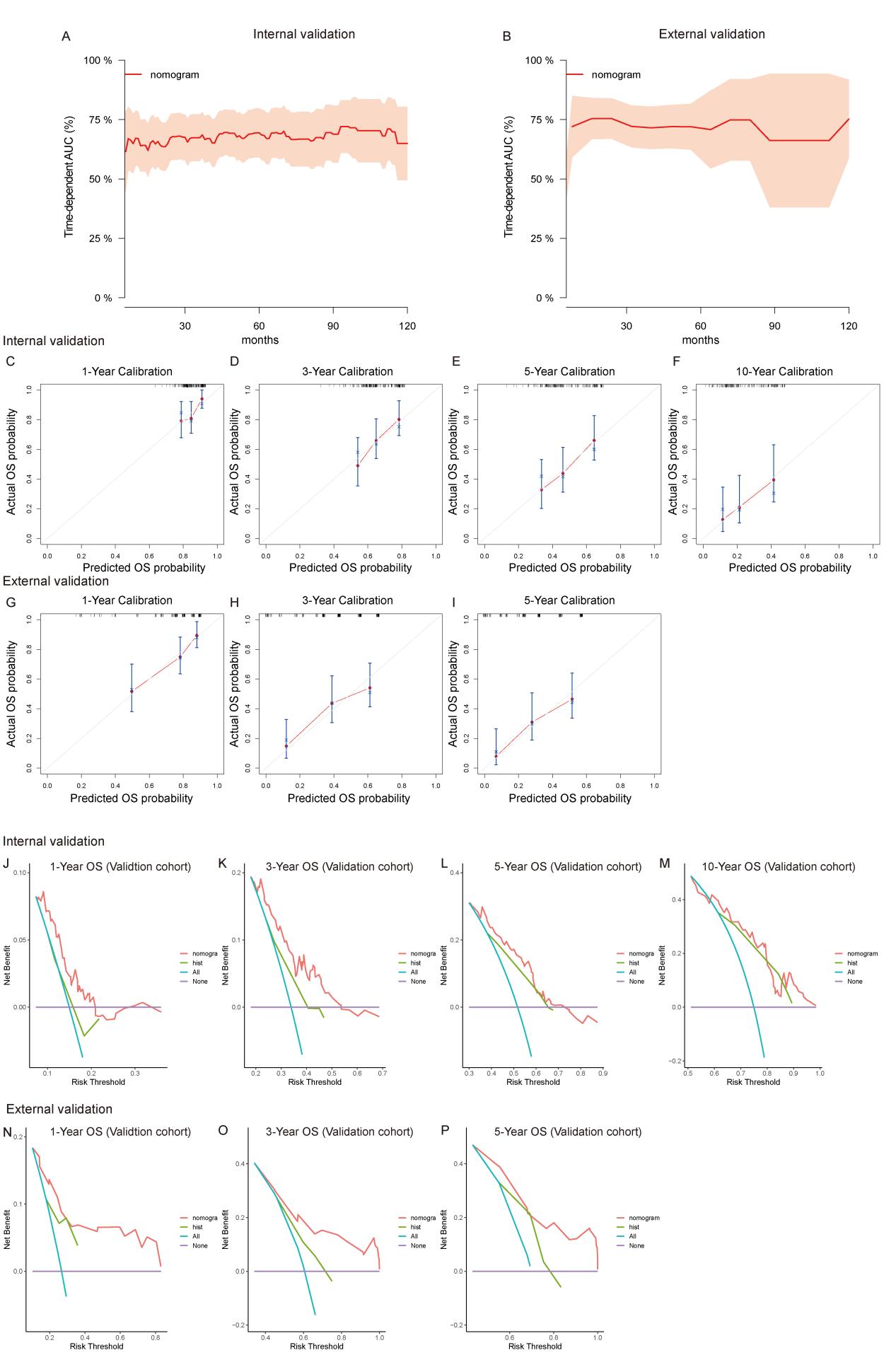


**Supplementary Figure 7**. The internal and external validation of the OS predictive nomogram for recurrent RLS. (A) Time-dependent AUC of using the nomogram to predict overall survival probability within 10 years in internal validation cohort. The 95% confidence interval was calculated by using the bootstrapping cross-validation method. (B) Time-dependent AUC of using the nomogram to predict overall survival probability within 10 years in external validation cohort. The 95% confidence interval was calculated by using the bootstrapping cross-validation method. (C-F) Calibration curves of 1-year, 3-year, 5-year and 10-year OS in internal validation cohort. (G-I) Calibration curves of 1-year, 3-year and 5-year OS in the external validation cohort. (J-M) Decision curve analysis of the nomogram and pathologic subtypes (hist) for the survival prediction within 10 years in internal validation cohort. (J-M) Decision curve analysis of the nomogram and pathologic subtypes (hist) for the survival prediction within 5 years in the external validation cohort.


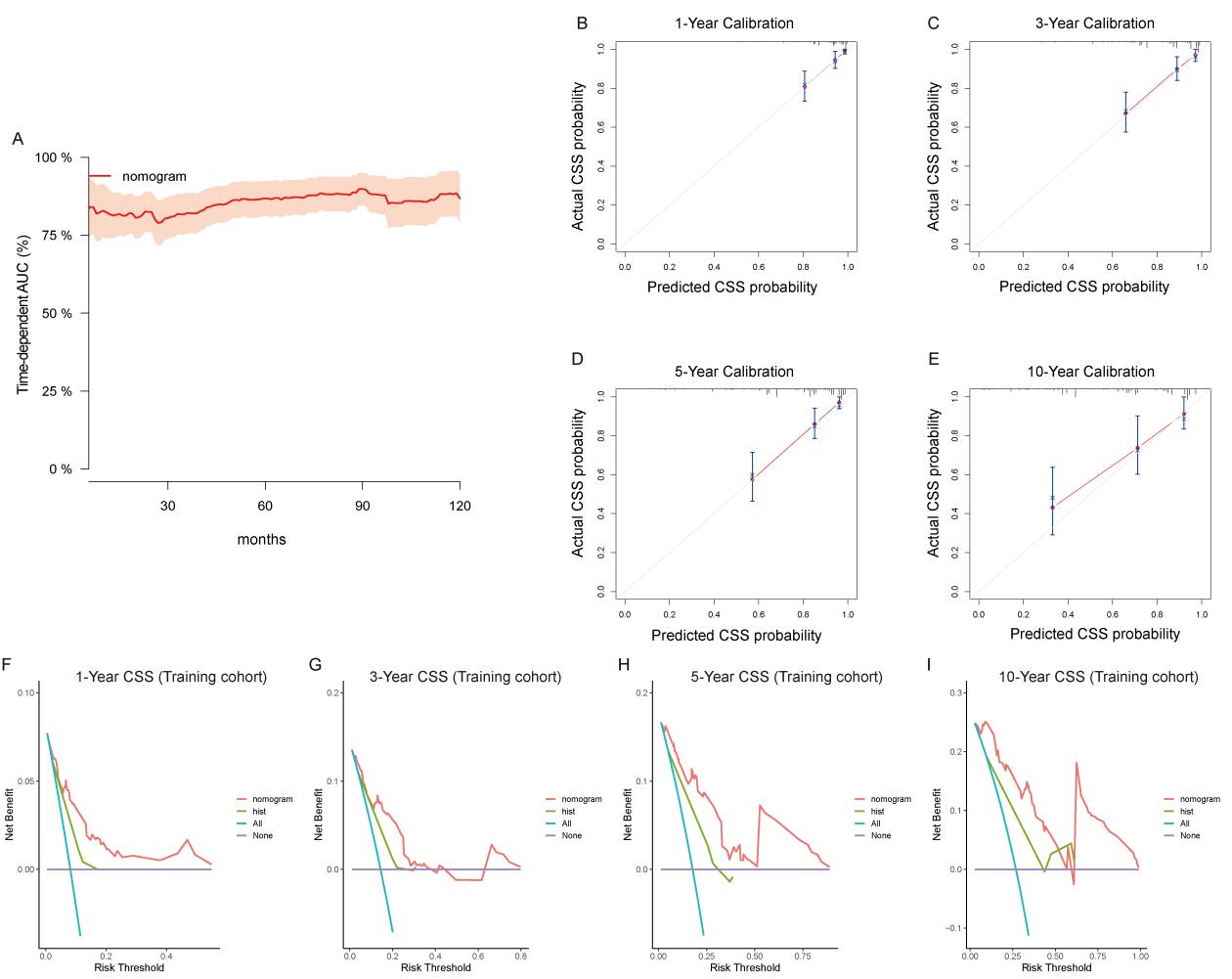


**Supplementary Figure 8**. The validation of the CSS predictive nomogram for recurrent RLS in the training cohort. (A) Time-dependent AUC of using the nomogram to predict CSS probability within 10 years in the training cohort. The 95% confidence interval was calculated by using the bootstrapping cross-validation method. (B-E) Calibration curves of 1-year, 3-year, 5-year and 10-year CSS in the training cohort. (F-I) Decision curve analysis of the nomogram and pathologic subtypes (hist) for the survival prediction within 10 years.


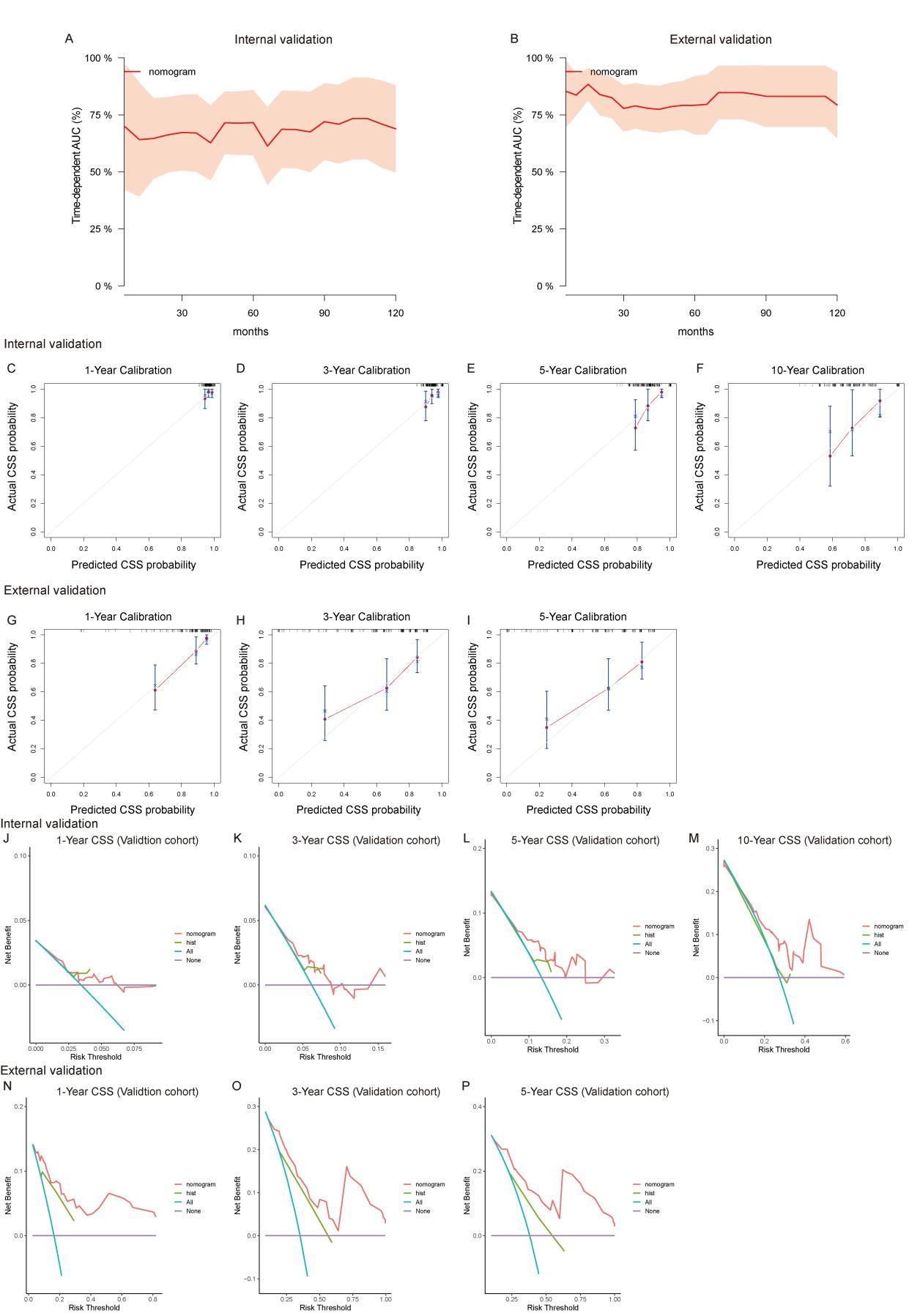


**Supplementary Figure 9**. The internal and external validation of the CSS predictive nomogram for recurrent RLS. (A) Time-dependent AUC of using the nomogram to predict CSS probability within 10 years in internal validation cohort. The 95% confidence interval was calculated by using the bootstrapping cross-validation method. (B) Time-dependent AUC of using the nomogram to predict CSS probability within 10 years in external validation cohort. The 95% confidence interval was calculated by using the bootstrapping cross-validation method. (C-F) Calibration curves of 1-year, 3-year, 5-year and 10-year CSS in internal validation cohort. (G-I) Calibration curves of 1-year, 3-year and 5-year CSS in the external validation cohort. (J-M) Decision curve analysis of the nomogram and pathologic subtypes (hist) for the survival prediction within 10 years in internal validation cohort. (J-M) Decision curve analysis of the nomogram and pathologic subtypes (hist) for the survival prediction within 5 years in the external validation cohort.
